# Ultrafast pore-loop dynamics in a AAA+ machine point to a Brownian-ratchet mechanism for protein translocation

**DOI:** 10.1126/sciadv.abg4674

**Published:** 2021-09-03

**Authors:** Hisham Mazal, Marija Iljina, Inbal Riven, Gilad Haran

**Affiliations:** Department of Chemical and Biological Physics, Weizmann Institute of Science, Rehovot 761001, Israel.

## Abstract

AAA+ ring–shaped machines, such as the disaggregation machines ClpB and Hsp104, mediate ATP-driven substrate translocation through their central channel by a set of pore loops. Recent structural studies have suggested a universal hand-over-hand translocation mechanism with slow and rigid subunit motions. However, functional and biophysical studies are in discord with this model. Here, we directly measure the real-time dynamics of the pore loops of ClpB during substrate threading, using single-molecule FRET spectroscopy. All pore loops undergo large-amplitude fluctuations on the microsecond time scale and change their conformation upon interaction with substrate proteins in an ATP-dependent manner. Conformational dynamics of two of the pore loops strongly correlate with disaggregation activity, suggesting that they are the main contributors to substrate pulling. This set of findings is rationalized in terms of an ultrafast Brownian-ratchet translocation mechanism, which likely acts in parallel to the much slower hand-over-hand process in ClpB and other AAA+ machines.

## Teaser

The pore-loop dynamics of the disaggregation machine ClpB are directly probed, pointing to a Brownian-ratchet mechanism of action.

## INTRODUCTION

**Fig. 1.**
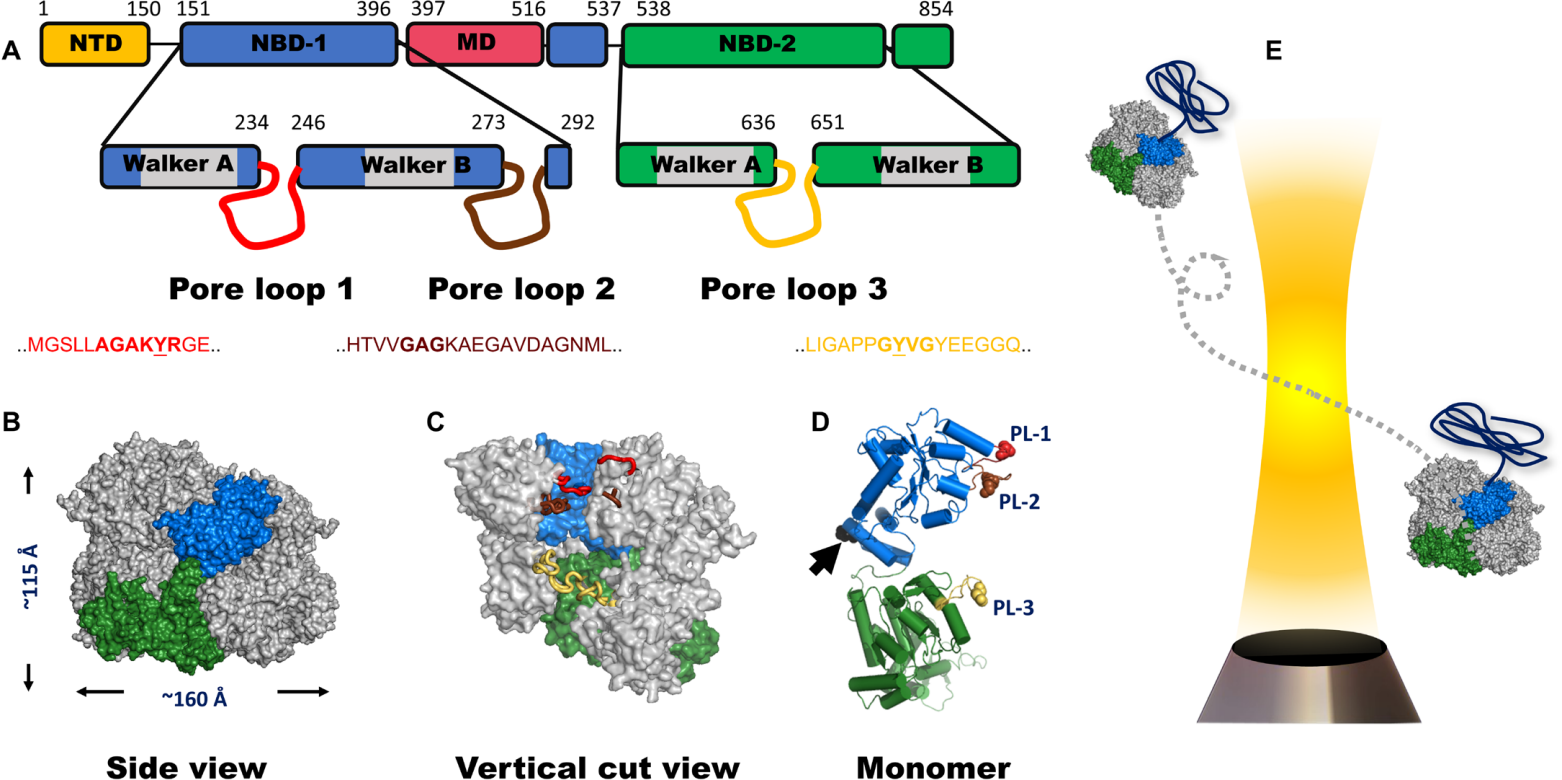
ClpB domain organization and structure. (**A**) Domain organization of the monomer of ClpB. NTD, N-terminal domain; NBD, nucleotide-binding domain; MD, middle domain. The location and sequence of pore loops are indicated on each NBD. The numbers mark residue positions in the sequence. A ClpB variant that lacks the NTD is used in this study. This variant actually occurs naturally and is fully functional. (**B**) Side view of the hexameric model of *E. coli* ClpB reconstructed from cryo-EM data (PDB: 6OAX). One protomer (protomer A) is highlighted using the same color code as in (A). The MD and the NTD are not resolved in this structure; thus, they are not shown. (**C**) Vertical cut view of the hexameric structure in (B). PL1, PL2, and PL3 of one protomer are colored in red, brown, and yellow, respectively. PL1 and PL2 are not fully resolved. (**D**) Monomeric structure of *E. coli* ClpB (PDB: 6OAX, protomer A) with α-helices represented as cylinders and pore loops shown in the same color as in (C). The spheres on pore loops represent the conserved tyrosine residues of PL1 (red) and PL3 (yellow) and the alanine residue of the motif GAG in PL2 (brown). Black arrow points to position P368, which is equivalent to position S359 in *T. thermophilus* ClpB, used in our smFRET experiments. (**E**) Freely diffusing molecules of ClpB, assembled with a single double-labeled protomer per hexamer (see Methods), emit bursts of photons as they pass through a focused laser beam, from which the FRET efficiency is calculated.

AAA+ [adenosine triphosphatases (ATPases) associated with various activities] proteins form an abundant family of biological machines that harness the energy of adenosine triphosphate (ATP) binding and hydrolysis to power cellular tasks such as protein unfolding, protein disaggregation, DNA helicase activity, DNA replication initiation, and cellular cargo transport (*1*, *2*). These machines typically assemble into hexameric ring complexes that envelope a large central channel (*3*). Pore loops lining the axial channel are essential elements for machine activity (*3*, *4*), and may exert force to pull substrates through the channel. The remarkable disaggregation machine ClpB and its eukaryotic analog Hsp104 consist of two nucleotide-binding domains (NBDs) per subunit. NBD1 contains two functional pore loops, PL1 and PL2, while NBD2 contains a single functional pore loop, PL3 (Fig. 1) (*5*–*8*). Both PL1 and PL3 harbor a conserved motif that involves a functionally important tyrosine residue, and their role in substrate pulling in a nucleotide-dependent manner has been demonstrated (*6*, *7*, *9*–*11*).

Recently, high-resolution structures of multiple AAA+ molecular machines have been solved by cryogenic electron microscopy (cryo-EM) (*3*, *12*–*15*), showing that these proteins form hexameric spirals (Fig. 1, B to D), rather than the fully symmetric hexameric rings described earlier (*16*). On the basis of these recurrent features, it has been proposed that substrate-polypeptide threading through the central channel of the machines proceeds in a sequential, hand-over-hand manner, facilitated by rigid-body movement of the protomers with a step size equivalent to 2 amino acids per ATP hydrolysis cycle (*17*, *18*). Because the ATP hydrolysis rate of these proteins is low, on the order of ~0.05 to 3.5 ATP molecules per second (*12*, *13*, *15*, *19*), the translocation rate is expected to be slow. However, a recent single-molecule force spectroscopy study showed that, remarkably, translocation by ClpB is extremely fast, ~240 to 450 amino acids per second, and occurs in bursts of 14 to 28 amino acids (*20*). Studies on ClpX (*21*) and ClpA (*22*) also demonstrated substrate translocation with step sizes larger than 2 amino acids. Furthermore, it was found that these machines were active in translocation even when several of their six subunits were rendered inactive (*23*, *24*). The strong disagreement between models based on structural studies and the results of real-time measurements is intriguing and hinders our understanding of the basis of substrate remodeling by these machines.

A molecular picture of the elusive pore-loop motions is critical for elucidating the translocation mechanism of such complex machines. In this work, we tackled this problem by directly observing the dynamics of pore loops in ClpB and their coupling along the axial channel, as well as their response to substrate translocation, using single-molecule fluorescence resonance energy transfer (smFRET) experiments (Fig. 1E) (*25*, *26*). Our hidden Markov model algorithm for photon-by-photon analysis, H^2^MM (*27*), allowed us to identify microsecond motions, which we could model in terms of two major conformational states for each pore loop. Tyrosine mutations in PL1 and PL3 exposed a correlation of the dynamics of these two pore loops with machine activity. Further, we observed differential response to ATP hydrolysis along the axial channel. These results could be rationalized in terms of a Brownian-ratchet mechanism for protein translocation by ClpB.

## RESULTS AND DISCUSSION

### Single-molecule studies of pore-loop dynamics

**Fig. 2.**
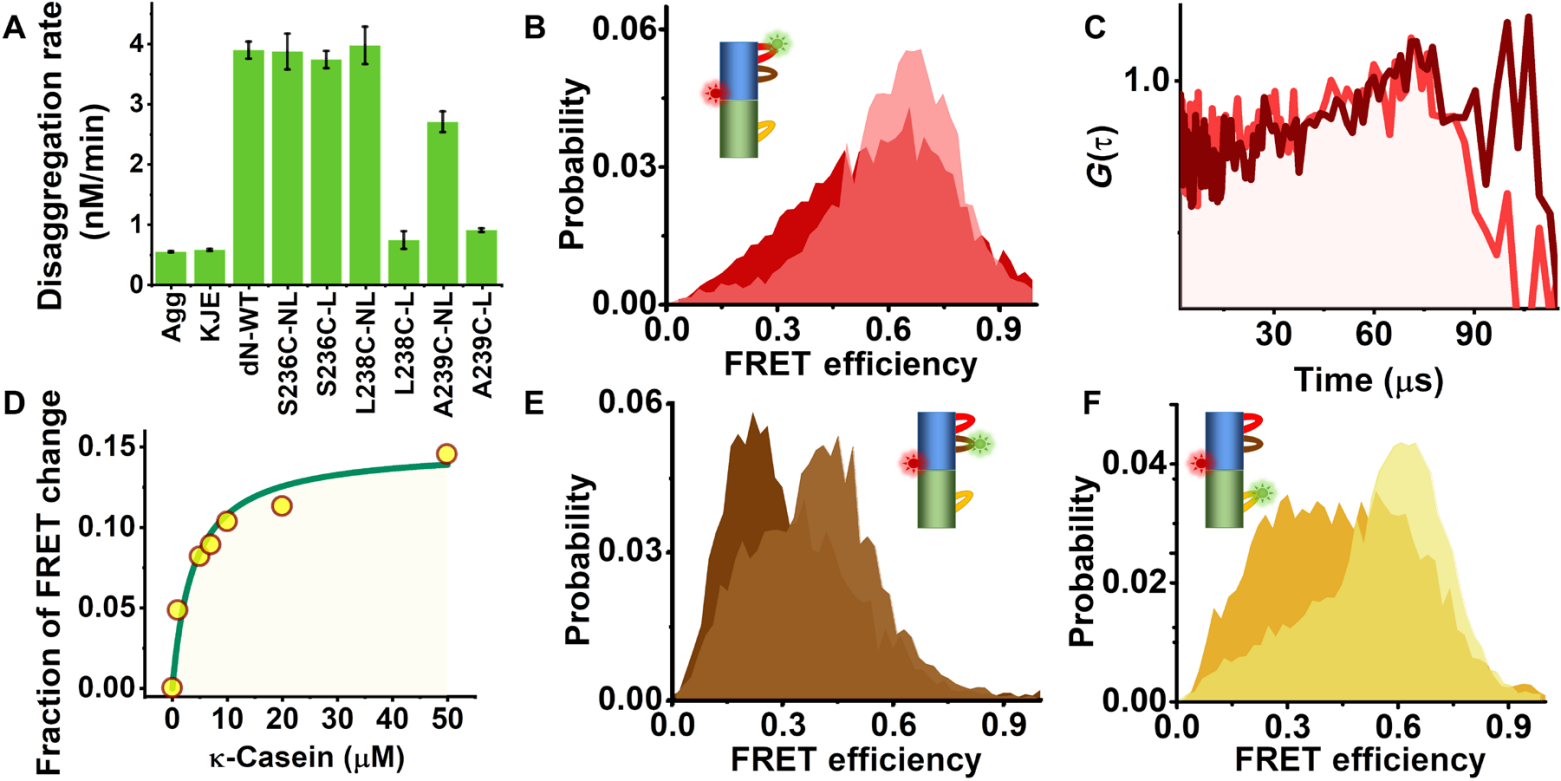
Probing pore-loop conformational dynamics using smFRET. (**A**) Disaggregation activity of ClpB WT and PL1 mutants. Results show that all nonlabeled pore-loop mutants (NL) were active, but only the mutant S236C maintained activity after labeling (L). Error bars were calculated from two experiments. (**B**) FRET-efficiency histograms of the PL1 variant without substrate (light red) and in the presence of 20 μM κ-casein (dark red). (**C**) Donor-acceptor fluorescence cross-correlation functions of the PL1 variant; light red, without substrate; dark red, with substrate. (**D**) Relative change of the low FRET-efficiency population in histograms (fig. S3A) as a function of κ-casein concentration. (**E**) FRET-efficiency histograms of the PL2 variant without substrate (light brown) and in the presence of 20 μM κ-casein (dark brown). (**F**) FRET-efficiency histograms of the PL3 variant without substrate (light orange) and in the presence of 20 μM κ-casein (dark orange).

We first studied PL1, which is located on NBD1 (Fig. 1D). To probe PL1 dynamics in its complete functional form, we mutated and labeled several residues flanking its conserved motif “AGAKYR” (Fig. 1A). We found that only the variant S236C maintained disaggregation activity after labeling (Fig. 2A, fig. S1, and table S1). We picked residue S359, located at the center of the vertical axis of the ClpB protomer, as a reference point to probe pore-loop motions along the axial channel (Fig. 1D). The same reference point was also used to study motion of the other pore loops. We thus prepared and labeled the variant S236C-S359C with donor (Alexa 488) and acceptor (Alexa 594) dyes and assembled ClpB molecules such that only a single subunit within each ClpB hexamer was labeled. Fluorescence anisotropy measurements (fig. S2, A to D, and table S2) ruled out motional restrictions of the fluorescent dye induced by interaction with ClpB’s channel or with substrate proteins (see below).

Photon-by-photon FRET trajectories were collected from labeled ClpB molecules freely diffusing through a focused laser beam in the presence of 2 mM ATP. The FRET-efficiency histogram constructed from the data (Fig. 2B and fig. S2E) showed a major population at a FRET-efficiency value of 0.65 ± 0.01. This major peak was found to be much broader than expected based on shot noise, indicating dynamic heterogeneity (see also fig. S2F). Donor-acceptor fluorescence cross-correlation curves calculated from the same data showed a rising component on a time scale of tens of microseconds, symptomatic of fast dynamics (Fig. 2C). To understand the role of PL1 in substrate engagement, we studied the effect of the soluble model substrate κ-casein (*19*, *28*). Enhanced ATPase activity of ClpB was measured in the presence of the substrate (fig. S1F), and its translocation was verified using the BAP-ClpP system (see Methods and fig. S1D). In the presence of 20 μM κ-casein, we noted a substrate concentration–dependent shift of the FRET-efficiency histograms toward lower values (Fig. 2, B and D, and fig. S3A), indicating a major conformational change, although no notable substrate-induced change in the dynamics was seen in the cross-correlation function (Fig. 2C).

To study PL2 dynamics in a similar manner (Fig. 1D and fig. S2, G and H), we mutated an alanine residue at position 281 to cysteine. The measured FRET-efficiency histogram of the double-labeled A281C-S359C mutant was markedly changed upon the addition of 20 μM κ-casein (Fig. 2E). The donor-acceptor fluorescence cross-correlation function again pointed to microsecond dynamics (fig. S3B). To study PL3, we mutated Y646 close to the conserved “GYVG” motif. The FRET-efficiency histogram of labeled S359C-Y646C in the presence of 2 mM ATP (Fig. 2F and fig. S2, I and J) changed substantially upon the addition of 20 μM κ-casein, with a significant increase in the low FRET-efficiency shoulder. Fluorescence cross-correlation analysis showed again evidence for fast dynamics (fig. S3C). We ruled out the involvement of NBD1-NBD2 interdomain motion in these dynamics (fig. S3, D to F).

**Fig. 3.**
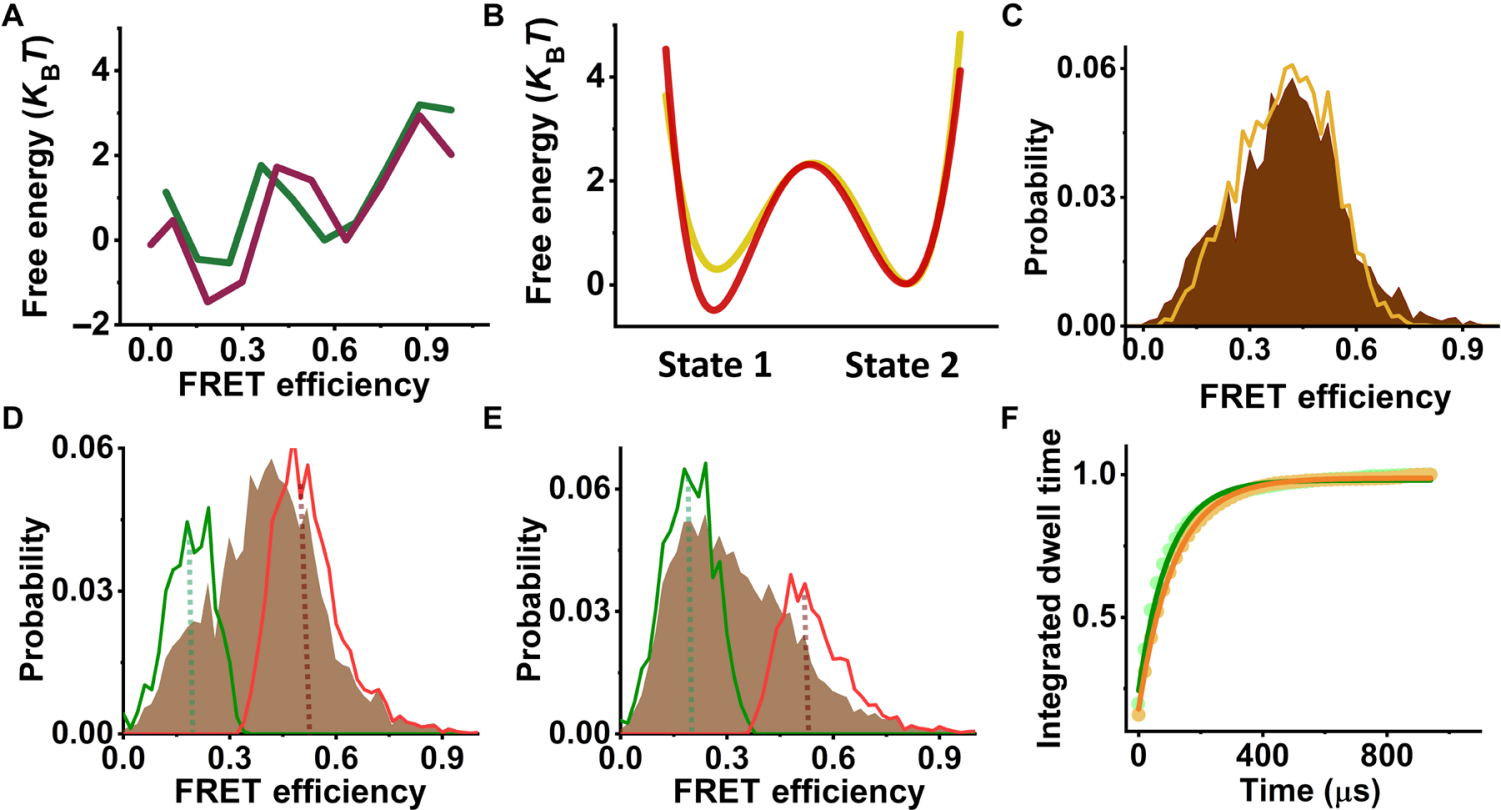
Analysis of PL2 conformational dynamics. (**A**) Free-energy profiles of PL2, as obtained from H^2^MM analysis. Pore-loop FRET data in the presence of 2 mM ATP and either without (green line) or with 20 μM κ-casein (purple line) were analyzed as discussed in Methods using nine sequentially connected states. Two well-defined minima are observed, indicating a two-state system. (**B**) Line plots of the effective free-energy profiles as obtained from a two-state H^2^MM from data measured either without (yellow line) or with 20 μM κ-casein (red line). The barrier heights in this figure were calculated using the Arrhenius equation with a pre-exponential factor of 10^5^ s^−1^. (**C**) A recolored FRET-efficiency histogram of PL2 (orange), obtained from analysis with a two-state model, matches well the original histogram (brown). (**D** and **E**) Segmentation analysis. Following the two-state H^2^MM analysis, segmented FRET-efficiency histograms were calculated according to the procedure outlined in Methods to obtain the distributions of the separate states. In each histogram, the green line is the low FRET-efficiency segmented population, and the red line is the high FRET-efficiency segmented population. Original histograms are plotted in brown. Dashed lines point to FRET-efficiency values of the two states obtained from the analysis. (D) With ATP only and (E) with ATP and 20 μM κ-casein. (**F**) Dwell time analysis. Integrated dwell-time distributions were calculated on the basis of the results of the two-state H^2^MM analysis as described in Methods. State 1 is shown as green dots, and state 2 is shown as orange dots. Green and orange solid lines are fits to single-exponential functions, and the obtained rates are listed in Table S3.

**Fig. 4.**
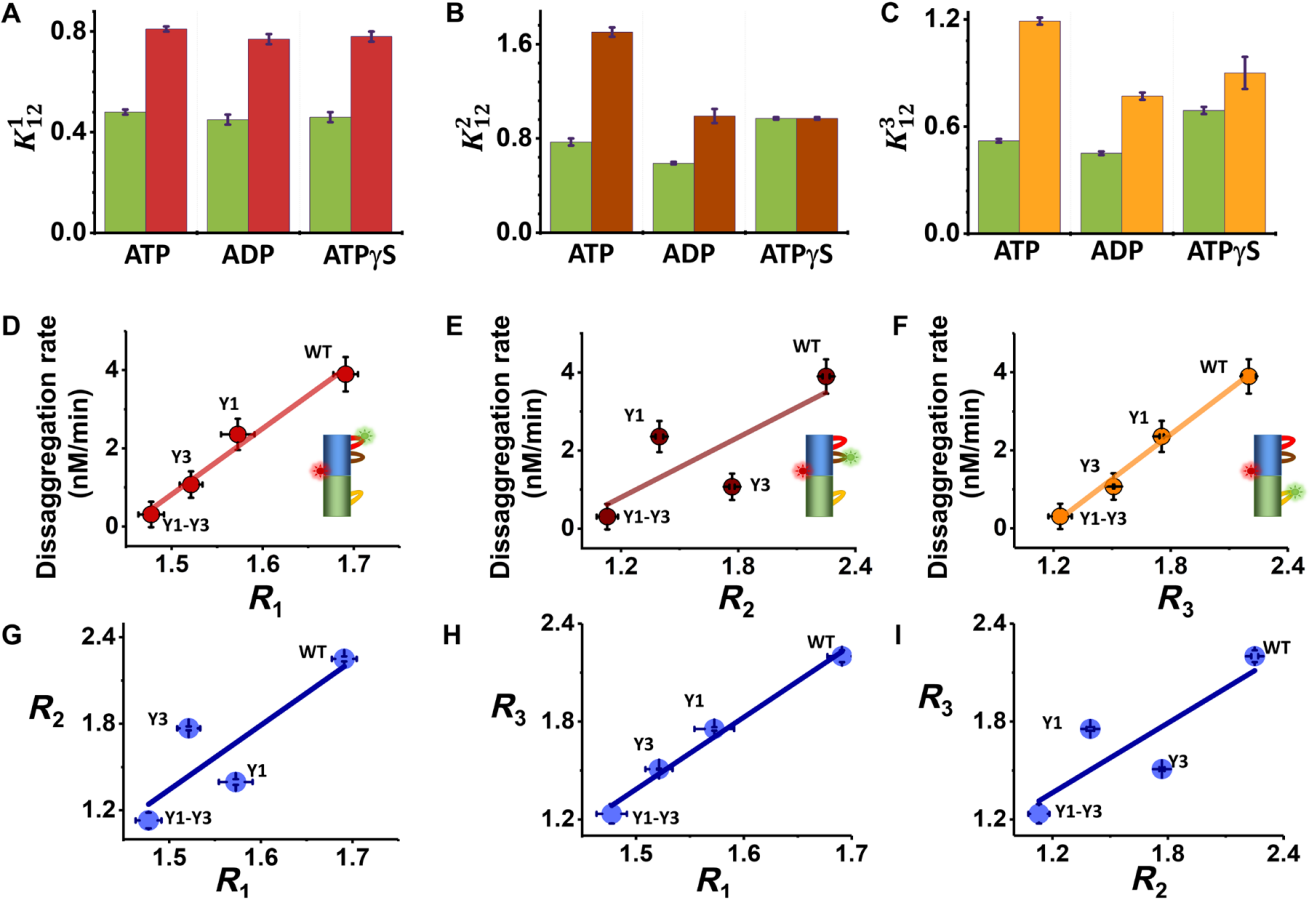
Modulating pore-loop conformational changes by nucleotides and tyrosine mutations. (**A** to **C**) Equilibrium coefficients for the conformational dynamics without or with 20 μM κ-casein and in the presence of different nucleotides (table S7). (A) PL1: green, no substrate; red, with substrate. (B) PL2: green, no substrate; brown, with substrate. (C) PL3: green, no substrate; yellow, with substrate. (**D** to **F**) Correlation of disaggregation rates (table S12) and the substrate-response factors, *R_i_*, of the three pore loops in tyrosine mutants (table S11). (**G** to **I**) Correlations between substrate-response factors of the three pore loops in tyrosine mutants. Errors were calculated from at least two experiments.

We initially attempted to model the dynamics of each pore loop in terms of a one-dimensional (1D) free-energy surface of an arbitrary shape. To this end, we analyzed the data assuming a Markov model involving a large number of sequentially connected states (*29*). The results of this analysis are shown in Fig. 3A for PL2 and in fig. S4 (A and B) for PL1 and PL3, respectively. Two well-defined potential wells, rather than a single minimum, were retrieved in each case, with microsecond time scale jumps between them. This outcome led us to represent the data using an effective two-state model. In particular, we globally analyzed pairs of datasets measured with and without κ-casein with a two-state model (Fig. 3B and fig. S4, C and D). The results (tables S3 to S5) were validated using several methods, including stochastic recoloring of data (Fig. 3C and fig. S5, A to E), segmentation analysis to reveal the two states in FRET-efficiency histograms (Fig. 3, D and E, and fig. S5, F to I), and dwell-time analysis (Fig. 3F and fig. S5, J to N). In particular, dwell times obtained from the latter analysis were in good agreement with transition rates directly obtained from the H^2^MM analysis of the two-state model (table S3). Using this two-state analysis, we obtained the population of each pore loop in its two states with and without the substrate (fig. S5, F to I, and table S5). An effective equilibrium coefficient for the conformational dynamics of pore loop *i*, $K_{12}^{i}$, was defined as its population ratio (table S7). Thus, for example, the population ratio in PL1, $K_{12}^{1}$, was 0.48 ± 0.01 and changed to 0.81 ± 0.01 in the presence of κ-casein (Fig. 4A). Further, we used the FRET-efficiency values of the two states obtained from the H^2^MM analysis, together with information from ClpB structures (table S4 and fig. S6A), to estimate the amplitude of motion of each pore loop. We calculated a motion of more than 10 Å in all cases, corresponding to as much as two substrate-protein residues. These large fluctuations, which could not be inferred from recent static high-resolution structures (fig. S6, B to D), are likely to contribute to substrate translocation on a much faster timescale than expected based on ATP hydrolysis rates.

### Nucleotides differentially affect pore-loop dynamics

To understand the mechanochemical coupling between ATP hydrolysis and pore-loop motions, we studied the effect of different nucleotides on the dynamics, including (in addition to ATP) adenosine diphosphate (ADP), to mimic the post-hydrolysis state and the slowly hydrolysable analog adenosine 5′-[γ-thio]triphosphate (ATPγS). Parameters derived from H^2^MM analysis of measured smFRET data are given in tables S5, S7, and S9. To quantify the effect of nucleotides on pore-loop conformational dynamics, we computed $K_{12}^{i}$ values from these parameters and then calculated the substrate-response factors, which are ratios of $K_{12}^{i}$ values with and without the substrate, $K_{12}^{i}$ (table S11). Unexpectedly, FRET-efficiency distributions of PL1 registered in the presence of ADP and ATPγS were almost identical to the equivalent histograms measured with ATP, either with or without κ-casein (fig. S7, A and D). Correspondingly, $K_{12}^{1}$ values (Fig. 4A) were very similar irrespective of the nucleotide used, and so were *R*_1_ values (table S11): 1.68 ± 0.01 with ATP and similar values with ADP and ATPγS. In contrast, PL2 demonstrated a differential response to nucleotides [histograms in fig. S7 (B and E), $K_{12}^{2}$ in Fig. 4B]. Accordingly, *R*_2_ changed from 2.25 ± 0.03 in ATP to 1.67 ± 0.02 in ADP, indicating a weaker response to the substrate. Remarkably, ATPγS did not elicit any substrate-induced change in the FRET-efficiency histogram of PL2, and *R*_2_ for this nucleotide was 1.00 ± 0.02.

In the case of PL3, the FRET-efficiency histograms (fig. S7, C and F) and $K_{12}^{3}$ values (Fig. 4C) also showed the largest response to substrate addition in the presence of ATP, with a corresponding *R*_3_ value of 2.28 ± 0.01, although a significant change was also registered in the presence of ADP (*R*_3_ = 1.71 ± 0.02) and a weaker response with ATPγS (*R*_3_ = 1.34 ± 0.02).

Together, these results indicate that ATP hydrolysis and likely the presence of the product P_i_ are important for a significant shift of the free-energy surfaces of PL2 and PL3 by the substrate, while PL1 is completely nucleotide-type independent.

### Correlation of dynamics to disaggregation activity

To probe the correlation of pore-loop conformational changes with machine activity, we investigated mutants of the conserved tyrosine residues located on PL1 and PL3. Several studies showed that mutation of these tyrosine residues to alanines resulted in reduced machine activity, with a more pronounced effect in the case of PL3 (*6*, *11*, *30*). We generated either a tyrosine mutant of PL1, Y243A (Y1), a tyrosine mutant of PL3, Y643A (Y3), or a double mutant, Y243A-Y643A (Y1-Y3). Glucose-6-phosphate dehydrogenase (G6PDH) disaggregation activity was reduced in all tyrosine mutants (fig. S1G and table S12) (*11*, *30*), with Y3 showing a stronger activity reduction than Y1, and Y1-Y3 showing almost no disaggregation activity. smFRET measurements were performed on each pore loop in constructs bearing Y1, Y3, or Y1-Y3 in the presence of 2 mM ATP and with or without 20 μM κ-casein (fig. S7, G to O) Parameters derived from H^2^MM analysis of these data are given in tables S6, S8, and S10. Correlation analysis of the disaggregation activity of each tyrosine mutant with substrate-response factors, *R_i_* (Fig. 4, D to F; see table S11 for the values of these parameters), demonstrated significant correlation for PL1 and PL3, with *R*^2^ values of 0.98 and 0.95, respectively. In contrast, PL2 demonstrated a weaker correlation, with an *R*^2^ value of 0.64. These results indicate that the conformational changes occurring in both PL1 and PL3 contribute significantly to machine activity in terms of protein translocation. The high correlation between PL3 dynamics and the overall disaggregation activity of the machine is in line with previous findings that NBD2 is the main contributor to machine activity (*19*, *31*), but the high correlation of PL1 dynamics to disaggregation is less anticipated. Notably, correlation plots between substrate-response factors of the different pore loops (Fig. 4, G to I) demonstrated a strong correlation between PL1 and PL3, with an *R*^2^ value of 0.99. All correlations observed here were validated through an additional calculation that did not depend on H^2^MM analysis of the data, as described in Methods.

### Brownian-ratchet model for protein translocation by ClpB

Our findings confer a more active role to pore-loop dynamics in machine activity than perceived before. The microsecond conformational dynamics of PL1 and PL3 not only are correlated with each other but also strongly correlate with the disaggregation rate of the machine and, therefore, also with substrate translocation rate. At the same time, both PL2 and PL3, but not PL1, seem to require nucleotide hydrolysis to change their conformation in response to substrate translocation, although their dynamics are much faster than ATP hydrolysis. In particular, our results suggest that the relative propensities of the two fast-exchanging states of PL2 and PL3 are modulated when the substrate is added and ATP hydrolysis has occurred. Our observations on PL2 are in line with a recent structural study of Hsp104 (*6*).

**Fig. 5.**
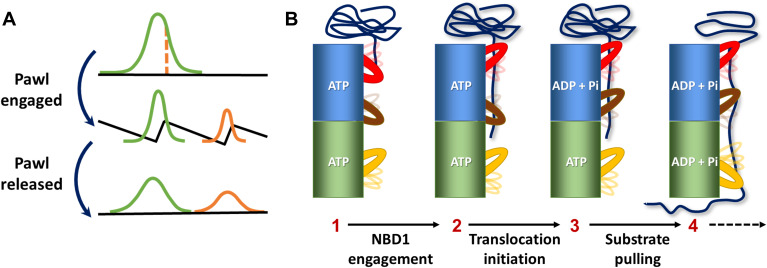
Brownian-ratchet mechanism for fast substrate translocation by ClpB. (**A**) In a Brownian ratchet, an effective pawl periodically switches molecular dynamics between a flat free-energy surface and a structured surface, promoting unidirectional motion. (**B**) Model for a potential Brownian-ratchet action of pore loops. As a substrate is engaged, pore loops gradually change their average conformation even while continuing to fluctuate on the microsecond time scale between two conformational states. The change in the population ratio of the two states of PL2 and PL3, which likely takes place only upon hydrolysis of ATP, is equivalent to a shift between two free-energy surfaces, as in (A), and turns them into effective pawls. At the same time, PL1 and PL3 function in pulling the substrate through the central channel.

These remarkable results point to a Brownian-ratchet–like mechanism (*32*–*34*) for fast substrate translocation by ClpB. In a typical Brownian-ratchet model (Fig. 5A), the input of chemical energy (e.g., ATP hydrolysis) transfers a machine between a state that allows free diffusion and a state with a pawl-like free-energy surface that rectifies the overall motion. The strong correlation between the structural changes of PL1 and PL3 and the disaggregation rate implies that these pore loops are active in pulling the substrate across ClpB’s channel (Fig. 5B). At the same time, the requirement for ATP hydrolysis for PL2 and PL3 conformational changes points to these pore loops as the ratchet pawls that operate to rectify substrate motion at different stages of translocation through the channel. PL2 may act first as a pawl when the substrate interacts with NBD1 and ATP has been hydrolyzed (Fig. 5B, step 3). This result is supported by a recent optical tweezers study of ClpA, which showed that ATP hydrolysis in NBD1 is important to prevent back-slipping of a client substrate (*22*). Similar events at NBD2 may then engage PL3 as a pawl (Fig. 5B, step 4). It is possible that disengagement of these pawls allows looped polypeptide segments to escape after partial threading (*20*, *35*). The protein harnesses the power of asynchronous pulling by neighboring subunits to generate rapid processive translocation events. The fast fluctuations of the pore loops allow them to reconfigure along a protein substrate, facilitating proper gripping and pulling and likely preventing premature stalling. This Brownian-ratchet mechanism is an appealing model for the operation of ClpB, although the structural component(s) responsible for asymmetric substrate translocation remain to be identified. The Brownian ratchet can operate in parallel to the much slower hand-over-hand process. Because pore loops are highly conserved in almost all AAA+ proteins (*3*, *36*, *37*), and because deviations from the predictions of the hand-over-hand mechanism have also been observed in several of them (*20*–*22*), it is likely that the Brownian-ratchet mechanism is a general mode of operation for these machines.

## METHODS

### Protein purification

The DNA of the *Thermus thermophilus* ClpB variant with a cleaved N-terminal domain, starting at residue 141 (ΔN-ClpB), mutants of this construct, the full-length ClpB variant modified to bind ClpP protease (BAP), and the cochaperones DnaK, DnaJ, and GrpE were cloned into a pET28b vector with the addition of a six-histidine tag preceded by a tobacco etch virus protease cleavage site, and purified by following our recently published protocol (*31*). ClpP was cloned in a pET9a vector with a C-terminal histidine tag and was also purified following the same purification procedure. Briefly, *Escherichia coli* BL21 (DE3) pLysS cells transformed with protein vectors were grown to OD (optical density) of 0.9 to 1.0 at 37°C. Expression was induced by adding 1 mM isopropyl-β-d-thiogalactopyranoside, and the cells were then incubated at 25°C overnight. Bacteria were then harvested, and the proteins were purified on a Ni–nitrilotriacetic acid resin (GE Healthcare) and dialyzed overnight in the presence of 1 mM ATP. Last, proteins were further purified using a HiPrep DEAE FF (GE Healthcare) column equilibrated with 50 mM Hepes, 20 mM KCl, and 2 mM tris(2-carboxyethyl)phosphine (TCEP) at pH 7.4 (DEAE buffer). The peak containing the purified protein was collected, and purity was assessed by gel electrophoresis (see fig. S1 for native gels and table S1 for sequence with marked mutation positions and for a complete list of the primers used in this study).

### Protein labeling

We took advantage of the physiologically relevant ClpB version with a truncated N-terminal domain (ΔN-ClpB, from here ClpB) (*38*–*40*), which allowed us to directly label the pore loops without any hindrance from this domain. ClpB labeling was carried out as described in our previous work (*31*). Double-cysteine mutants were exchanged into labeling buffer 1 (25 mM Hepes and 25 mM KCl, pH 7) and then reacted with the acceptor dye molecules, Alexa 594 C5 maleimide, at a 1:1.2 protein to dye ratio for 2 hours. Protein molecules were then separated from the unreacted dye using a desalting column (Sephadex G25, GE Healthcare), equilibrated with the same labeling buffer but including 2 M guanidinium chloride (GdmCl) to expose the partially buried sites for labeling. ClpB in 2 M GdmCl was then reacted with the donor dye Alexa 488 C5 maleimide at a 1:0.8 protein to dye ratio for 2 hours. Unreacted dye molecules were then separated using a desalting column equilibrated with the labeling buffer including 2 M GdmCl. To obtain ClpB hexamers with a single labeled protomer or less, we reassembled ClpB molecules by mixing labeled subunits with 100-fold molar excess of cysteine-less subunits according to the mixing procedure described below.

It is important to mention that in the case of the variant S236C-S359C, both sites are well exposed and might be labeled with donor and acceptor. On the other hand, in the case of A281C-S359C and S359C-Y646C, there is semi-specific labeling. In these mutants, S359C is labeled with the acceptor dye and the sites A281C and Y646C are labeled with the donor.

### Subunit mixing

To ensure that we obtain homogeneous mixing of the labeled and unlabeled ClpB subunits, we mixed them in a 6 M GdmCl solution, which contained 50 mM Hepes and 150 mM NaCl (the mixing buffer). Subsequently, the denaturant concentration was reduced step by step using dialysis, as follows: after the initial 4 hours in 6 M GdmCl, the buffer was exchanged and the sample was incubated until full equilibration was reached in the mixing buffers containing 4 M GdmCl, then 2 M GdmCl, 1 M GdmCl, and, finally, 0 M GdmCl. At each step, we confirmed that our samples reached equilibrium with the dialysis buffer using refractometry of the solution. Last, the mixed subunits were further extensively dialyzed against the smFRET buffer (25 mM Hepes, 25 mM KCl, 10 mM MgCl_2_, and 2 mM ATP, pH 7.8) to ensure their full refolding and reassembly. The 1:100 ratio of labeled to nonlabeled ClpB molecules was verified by comparing the absorbance of Alexa 488 at 490 nm and the protein at 280 nm. While in general we mixed double-labeled subunits with wild-type ΔN-ClpB subunits, in the case of ClpB molecules that contained a functional mutation (Y243A, Y643A, and Y243A-Y643A), the double-labeled subunits were mixed with cysteine-less variants of the same mutants. Native gel electrophoresis, gel filtration chromatography, enzymatic activity assays, and inter-subunit FRET measurements showed that the mixed labeled ClpB molecules were assembled and active (fig. S1). The assembled molecules were then filtered through 100-nm filters (Whatman Anotop-10), aliquoted, and stored at −80°C.

### ATPase activity

ATPase activity of ClpB was measured using a coupled colorimetric assay (*31*). ClpB (1 μM) (desalted to 25 mM Hepes and 25 mM KCl, pH 7.8) was incubated in the presence of various amounts of ATP (50 μM to 3 mM) in 25 mM Hepes (pH 7.8), 25 mM KCl, 0.01% Tween 20, and an ATP regeneration system [2.5 mM phosphoenol pyruvate, pyruvate kinase (10 U/ml), lactate dehydrogenase (15 U/ml), 2 mM 1,4-dithioerythritol, 2 mM EDTA, and 0.25 mM NADH (reduced form of nicotinamide adenine dinucleotide)] at 25°C. The reaction was started by adding MgCl_2_ to a final concentration of 10 mM. ATP hydrolysis rate was then measured indirectly by monitoring NADH absorption at 340 nm over time using a microplate reader (Synergy HTX, BioTek). The rate at each ATP concentration was determined from the initial slope of the reaction and was background-corrected. It was then plotted as a function of ATP concentration and fitted to the Hill equation

$$\nu =V_{\text{max}}\frac{S^{n}}{S^{n}+K_{0.5}^{n}}$$

where ν is the initial reaction velocity, *V*_max_ is the maximum reaction velocity at saturating substrate concentration, *S* is the substrate concentration, *K*_0.5_ is the concentration at which half of the molecules are bound, and *n* is the Hill constant. To test the stimulation of ATP hydrolysis of ClpB, we incubated ClpB with κ-casein at target concentrations of 0 to 50 μM, using the same reaction buffer as above. ATP hydrolysis rate was calculated as described above (fig. S1, E and F, and table S12).

### Protein disaggregation activity

This assay was carried out as described previously (*31*). Briefly, 90 μM G6PDH from *Leuconostoc mesenteroides* was denatured by heating at 47°C for 5 min in a buffer containing 50 mM Hepes, 5 M urea, 20 mM dithiothreitol, and 7.5% glycerol. Aggregate formation was achieved by diluting (1:100) the denatured G6PDH into a reactivation buffer [50 mM Hepes, 30 mM KCl, 1 mM EDTA, 1 mM TCEP, 3 mM ATP, pyruvate kinase (20 μg/μl), 3 mM phosphoenol pyruvate, and 20 mM MgCl_2_] followed by heating at 47°C for 10 min. G6PDH aggregates (750 nM) were mixed with 2 μM ClpB, 4 μM DnaK, 1 μM DnaJ, and 1 μM GrpE in the same reactivation buffer as before, and this disaggregation reaction mixture was incubated at 37°C. The recovered activity of G6PDH protein was then measured at different time points during the incubation, from which the rate of disaggregation was calculated, exactly as described before (fig. S1G and table S12) (*31*).

### Protein degradation activity

To verify that ClpB can translocate κ-casein, we prepared a BAP variant of ClpB (*41*), which can bind to ClpP and transfer substrates into ClpP for degradation. The degradation activity assay was carried out as described previously (*42*). κ-Casein (20 μM) was incubated with 2 μM BAP, 2.5 μM ClpP, 4 μM DnaK, 1 μM DnaJ, and 1 μM GrpE, in a buffer containing 25 mM Hepes, 25 mM KCl, 10 mM MgCl_2_, and 2 mM TCEP (pH 7.8). The reaction solution was incubated at 37°C for up to 8 hours. Twelve microliters from each sample were withdrawn every few hours during the incubation, and the reaction was quenched by mixing with SDS gel sample buffer and heating at 95°C for 5 min. Last, κ-casein concentrations were quantified by SDS gel electrophoresis. Results showed that κ-casein was degraded in the presence of the BAP-ClpP construct, indicating translocation of κ-casein (fig. S1D).

### Sample preparation and smFRET experiments

Samples and flow cells were prepared exactly as described in our previous publication (*31*). Single-molecule measurements were conducted on freely diffusing molecules, using a MicroTime 200 fluorescence microscope (PicoQuant). To sample both donor and acceptor dyes, we used pulsed interleaved excitation with 485- and 594-nm diode lasers pulsed at a ratio of 3:1 at a repetition rate of 40 MHz and laser power of 50 and 10 μW for each laser, respectively. The emitted photons were divided into two channels according to their wavelengths using a dichroic mirror (FF580-FDi01; Semrock) and filtered with band-pass filters (520/35 nm, BrightLine, Semrock, for the donor channel and ET-645/75 m, Chroma, for the acceptor channel). Arrival times of these photons were registered by two single-photon avalanche photodiodes (Excelitas SPCM-AQR-14-TR) coupled to a standalone time-correlated single-photon counting module (HydraHarp 400, PicoQuant).

We detected fluorescence bursts in the single-molecule data using methods developed in the laboratory and described in previous studies (*31*, *43*, *44*). Fluorescence bursts were separated from the background photons using a cutoff time of 5 μs, and only fluorescence bursts with a total of 30 photons or more were selected for further analysis. The raw FRET efficiency of each burst was then calculated on the basis of the photons detected in both channels after donor excitation only, and the raw stoichiometry was calculated from the detected photons in both channels after both excitations. A 2D histogram of raw stoichiometry versus raw FRET efficiency was generated (fig. S2), from which we calculated the amount of emitted donor photons leaking into the acceptor channel, and the level of direct excitation of the acceptor dye by the 485-nm laser. We corrected the photon stream in both channels based on the calculated correction factors. To remove the donor-only and acceptor-only populations from the FRET-efficiency histograms, we selected only photon bursts with a stoichiometry that corresponded to double-labeled molecules, with both donor and acceptor dyes (fig. S2, E, G, and I). Following the selection of double-labeled molecules, we selected their photons arising from donor excitation only and analyzed these photons using burst variance analysis (*45*) (fig. S2, F, H, and J), fluorescence correlation spectroscopy (FCS), and H^2^MM (see below) to characterize and quantify the fast dynamics.

It has been suggested, on the basis of structural models (*3*), that at each ATP hydrolysis cycle of ClpB, there are four to five protomers engaged with the substrate and one to two protomers disengaged. Thus, our results are more likely (~83%) to represent the four to five engaged protomers and are less likely to represent the so-called seam protomer (~17%). Furthermore, our observation time per molecule was ~1 ms, which is way shorter than ATP hydrolysis (fig. S1F), so that changes in the ATP status of protomers during fluorescence burst measurements were very rare.

### Per-burst FCS calculation

Cross-correlation functions of the donor and acceptor fluorescence on a burst-by-burst basis were calculated similarly to previously published methods (*46*). For the calculation, only the double-labeled species were selected (fig. S2). An increase in the cross-correlation at early times indicated fast conformational dynamics (Fig. 2C and fig. S3, B and C), while no increase indicates no detectable dynamics (fig. S3F).

### Fluorescence anisotropy measurements

Time-resolved fluorescence anisotropy experiments (fig. S2, A to D) were conducted on target proteins using a MicroTime200 fluorescence microscope (PicoQuant). Samples were diluted to 1 to 2 nM in the working buffer (25 mM Hepes, 25 mM KCl, 10 mM MgCl_2_, and 2 mM ATP, with and without 20 μM κ-casein) containing 0.01% Tween 20 and then loaded into a flow cell precoated with a lipid bilayer (*31*). Molecules were excited with a 485-nm diode laser pulsed at a repetition rate of 40 MHz with a power of 50 μW. Emitted photons were passed through a 50-μm pinhole and divided on the basis of their polarization using a polarizing beam splitter cube, followed by filtration using band-pass filters (520/35 nm, BrightLine, Semrock). Photon arrival times relative to the excitation pulse were registered using two single-photon avalanche photodiode detectors (Excelitas SPCM-AQR-14-TR) coupled to a time-correlated single-photon counting module (HydraHarp 400, PicoQuant). The parallel and perpendicular fluorescence decays were constructed from the data and background-corrected. Fluorescence anisotropy decays were then calculated using the following relation: $r(t)=\frac{I_{∥(t)}-{\mathrm{GI}}_{⊥(t)}}{I_{∥(t)}+2{\mathrm{GI}}_{⊥(t)}}$, where *I*_∥(*t*)_ and *I*_⊥(*t*)_ are the time-dependent fluorescence intensities of the parallel and perpendicular components, and *G* is the polarization sensitivity factor (whose value was determined to be 1).

Steady-state fluorescence anisotropy values were measured using a Horiba Jobin-Yvon Fluorolog fluorimeter, and these values are provided in table S2. Steady-state fluorescence anisotropy values were also calculated from the time-resolved data using the formula $r=\frac{\sum I_{∥(t)}-G\sum I_{⊥(t)}}{\sum I_{∥(t)}+2G\sum I_{⊥(t)}}$ and are also listed in table S2.

While the steady-state anisotropy values of pore-loop labels are relatively high, an observation of the anisotropy decay curves suggests that these values reflect the overall slow tumbling of the ClpB hexamer, while at short times a fast anisotropy decay uniformly appears in the data. No significant changes in either anisotropy decay curves or steady-state values were registered upon addition of substrate proteins, indicating the absence of motional restriction by the substrate.

### Comparison of single molecule–derived and cryo-EM–predicted FRET distributions

To compare our FRET data to cryo-EM derived data, we calculated a list of distances between the residues labeled in this work in pore loops of all available ClpB structures. In particular, we used Protein Data Bank (PDB) files 6OG1, 6OAX, 6OAY, 6RN2, 6RN3, 6RN4, 6QS6, 6QS7, SQS8, 5OG1, and 5OF1 to map all possible distances of these pore loops, irrespective of the conditions used. We then calculated the expected FRET-efficiency values for these distances, using a measured value for the Förster distance (54 Å) (*31*). Results are shown in fig. S6.

### H^2^MM analysis

The algorithm H^2^MM for photon-by-photon smFRET data analysis was introduced in (*27*). The Matlab code for H^2^MM can be found at https://pubs.acs.org/doi/abs/10.1021/acs.jpcb.6b10726. The inputs for the analysis are the arrival times of the donor and acceptor photons, which constitute the observation sequence (*O*). A hidden Markov chain is specified by the following components:

1) Prior matrix (Pi) (Π), Π = [π_1_,_2_….π*_n_*], which is a stochastic matrix and defines the probability of a Markov chain to start with state *x* out of *n* states.

2) A transition matrix, $A=(\begin{array}{ccc} a_{11} & \ldots & a_{1n}\\ \ldots & \ldots & \ldots \\ a_{n1} & \ldots & a_{\mathrm{nn}} \end{array})$,

which is also a stochastic matrix that includes all the probabilities to move between pairs of states (from *x* to *y*) out of the *n* available ones.

3) The observation matrix, $B=(\begin{array}{ccc} b_{11} & \ldots & b_{1m}\\ \ldots & \ldots & \ldots \\ b_{n1} & \ldots & b_{\mathrm{nm}} \end{array}),$

where *m* defines the number of observables and *n* defines the number of states. For simple two-color FRET experiments on the photon-by-photon level, there are only two observables, the donor and acceptor photons (*m* = 2).

The algorithm run proceeds according to the following steps:

i) Initialization: Guess initial model parameters *A*, *B*, and Π. (Here, we use 50 to 100 initial guesses.)

ii) Expectation: Given an observation sequence *O* and the set of states in the HMM, we learn the HMM parameters Π, *A*, and *B*. This step is done iteratively using the forward-backward algorithm (Baum-Welch algorithm), a special case of the Expectation-Maximization algorithm.

iii) Maximization: Re-estimate Π, *A*, and *B* based on estimators calculated in the previous step.

iv) Determine the likelihood *P*(*O*|λ); simply, what is the probability that the observations are generated by the model. If the likelihood converged, then end and output the model; otherwise, repeat the cycle from ii.

v) Viterbi algorithm: Using the best model λ = (П, *A*, *B*), calculate the most probable sequence of states. Here, we take the best model (highest likelihood) from all the 50 to 100 initial guesses and then run the Viterbi algorithm for this model.

In general, we choose ~10,000 molecules (as described above) from each dataset for analysis. In case of a “free model,” we do not restrict any model parameters λ = (Π, *A*, *B*). In case of global analysis of the data with and without the substrate, we let the model parameters Π and *A* to be optimized freely and independently; however, the observation matrix is optimized from the calculation of two datasets together.

### Validation of H^2^MM analysis

We validated the statistical analysis using the same procedures as outlined in (*31*). To “recolor” FRET-efficiency histograms, we kept the arrival times of photons in real experimental data fixed but erased their color identity (i.e., whether they belong to the donor or acceptor channel, green or red). We then recolored the photons using a Monte-Carlo simulation based on the model parameters obtained from H^2^MM analysis. For each burst, we calculated the average FRET efficiency and plotted a new histogram to compare to the original experimental histogram. In segmentation analysis, we used the most probable sequence of states in each trajectory (burst) as obtained from the H^2^MM analysis together with the Viterbi algorithm. We then calculated histograms based on the assignment of each segment to either state 1 or 2. Correct modeling should lead to separation of the two states in these histograms. Dwell-time distributions were calculated on the basis of the results of the two-state H^2^MM analysis, according to the procedure described in (*44*).

### Dynamics on a 1D free-energy surface modeled with H^2^MM

To represent pore-loop dynamics with a single continuous free-energy profile, we used a model of 9 to 10 equally spaced and sequentially connected states. To this end, we restricted the H^2^MM parameters as follows:

1) Observation matrix: we assumed a continuous number of states, rather than a fixed small number of states. Thus, we discretized the FRET reaction coordinate with 9 to 10 states with FRET-efficiency values equally distributed in the range from 0.05 to 0.98.

2) Transition probability matrix: we allowed sequential transitions only, namely, all transitions *a_ij_* that fulfil *i* = 1:*N* and *j* = *i* ± [0:1]. All the rest were set to zero.

3) Prior matrix (Π): no restrictions were set on the prior matrix.

Following these restrictions, we analyzed the data with H^2^MM model using 50 random initial guesses. We then adopted the results from the run that converged to the highest likelihood. The free-energy profile was then calculated from the occupancy of the states as follows: *V*(*E*) = −*K*_B_*T* ln (probability of the state) (fig. S4, A to C).

### Validation of correlation analyses (Fig. 4)

We validated the correlations shown in Fig. 4 using a method that did not depend on the H^2^MM analysis. To this end, we split each FRET-efficiency histogram into two different regions, a low FRET-efficiency region and a high FRET-efficiency region. The splitting point was determined on the basis of the intersection point of the low and high FRET-efficiency states as obtained from segmentation analysis (fig. S5, F to I). We calculated areas below and above the splitting point and used them to obtain new values for the equilibrium constants and for the substrate-response factors. Using these values to plot correlations as in Fig. 4 (D to I), we obtained very similar results, with disaggregation activity strongly correlated with *R*_1_ and *R*_3_, and, in turn, a strong correlation of these two substrate- response factors.

## Supplementary Materials

### This PDF file includes:

Figs. S1 to S7 Tables S1 to S12 References
