# Supplementary material for: Ultrafast pore-loop dynamics in a AAA+ machine point to a Brownian-ratchet mechanism for protein translocation
Source: Sci Adv. 2021 Sep 3;7(36):eabg4674. doi: 10.1126/sciadv.abg4674 (PMC8442866; doi:10.1126/sciadv.abg4674)
Supplement: Supplementary file 1 — Figs. S1 to S7 Tables S1 to S12 References [file sciadv.abg4674_sm.pdf]

Science Advances
AAAS

Supplementary Materials for

Ultrafast pore-loop dynamics in a AAA+ machine point to a Brownian-ratchet mechanism for protein translocation

Hisham Mazal, Marija Iljina, Inbal Riven, Gilad Haran*

*Corresponding author. Email: gilad.haran@weizmann.ac.il

Published 3 September 2021, *Sci. Adv.* **7**, eabg4674 (2021)
DOI: 10.1126/sciadv.abg4674

This PDF file includes:

Figs. S1 to S7
Tables S1 to S12

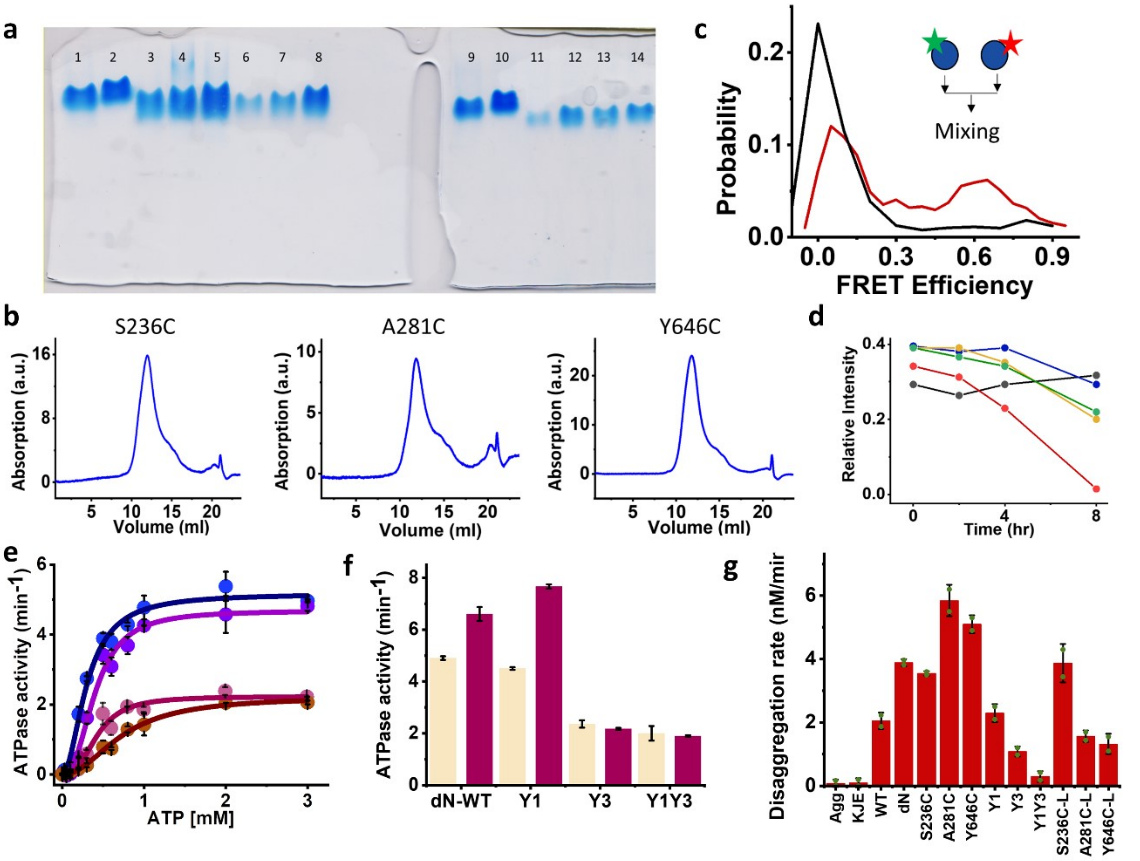

Fig. S1. Characterization of ΔN-ClpB mutants. (a) Native gel electrophoresis (3 % acrylamide), run in the presence of 10 mM $MgCl_2$ and 2 mM ATP. (1) Full length (FL) WT *TT* ClpB, (2) FL WT *TT* ClpB S428C–S771C, double-labeled with Alexa 488 and Alexa 594, and mixed 1:100 with cysteine-less FL ClpB. (3) Double-labeled ΔN-ClpB A281C-S359C mixed 1:100 with WT ΔN-ClpB. (4-5) Repeats of double-labeled ΔN-ClpB S359C-Y646C mixed 1:100 with WT ΔN-ClpB. (6) Double-labeled ΔN-ClpB S359C-Y646C-Y243A mixed 1:100 with ΔN-ClpB Y243A mutant. (7) Double-labeled ΔN-ClpB S359C-Y646C-Y643A mixed 1:100 with ΔN-ClpB Y643A mutant. (8) Double-labeled ΔN-ClpB S359C-Y646C-Y243A-Y643A mixed 1:100 with ΔN-ClpB Y243A-Y643A mutant. (9) FL WT *TT* ClpB, same as (1). (10) Same as 2. (11) Double-labeled ΔN-ClpB S236C-359C mixed 1:100 with WT ΔN-ClpB. (12) Double-labeled ΔN-ClpB S236C-S359C-Y243A mixed 1:100 with ΔN-ClpB Y243A mutant. (13) Double-labeled ΔN-ClpB S236C-S359C-Y643A mixed 1:100 with ΔN-ClpB Y643A mutant. (14) Double-labeled ΔN-ClpB S236C-S359C-Y243A-Y643A mixed 1:100 with ΔN-ClpB Y243A-Y643A mutant. The gel was stained with Coomassie Blue (a). We only observed one band in each case, indicating homogeneity of the samples. (b) Size-exclusion chromatography of labeled and assembled constructs. ΔN-ClpB single cysteine mutants PL1 (S236C), PL2 (A281C) and PL3 (Y646C) were purified and fully labeled with Alexa 594. The proteins were run on a SEC column (Superdex 200, 10/300 GL, total volume 23.6 ml) to qualitatively determine their assembly. ClpB molecules were diluted in 25 mM HEPES, 25 mM KCl, 10 mM $MgCl_2$ and 2 mM ATP. The labeled ClpB molecules eluted within the narrow peak at ~ 12 ml, confirming their assembly. As a reminder, in our single-molecule experiments, we mixed the labeled molecules in 1:100 ratio with non-labeled ClpB to obtain hexamers with only a single labeled protomer. Thus, ClpB hexamers should be assembled even better as there would be

no steric clashes between labeled protomers. (c) Validation of ClpB integrity under the smFRET experimental conditions. This experiment was done similarly to the previously published protocol (*31*). Here, ClpB single cysteine mutant (S236C) was purified, assembled and labeled with either Alexa 488 (donor) or Alexa 594 (acceptor), separately. We then mixed the donor and acceptor variants of each labeled mutant at a 1:1 ratio, and at a final concentration of 5 nM (inset). The mixing process was done using 6 M guanidinium chloride (GdmCl) followed by dialysis to 0 M GdmCl. We split the samples into two test tubes, one containing 2 mM ATP, and the other without ATP. We then performed smFRET experiments, to measure FRET-efficiency distribution as an indication of assembly. Black curve is S236C without ATP, which shows no high FRET-efficiency population, indicating the absence of assembly. Red curve is S236C with 2 mM ATP, showing a high FRET-efficiency population, an indication of assembly. The large peak at low FRET efficiency represents donor-only molecules. (d) Degradation of κ-casein by BAP and ClpP, performed according to the protocol in Ref. (*42*), except that the reaction was performed at 25°C rather than 55°C. The concentration of each component was as follows: 20 μM κ-casein, 2 mM ATP, 2 μM BAP and 2.5 μM ClpP, 4 μM DnaK, 1 μM DnaJ and GrpE. The reaction, containing different components, was let to proceed for 8 h. Samples at different time points were analyzed using SDS-PAGE, and the band intensity of κ-casein was measured using Image J. Black line is κ-casein alone. Blue line is κ-casein and BAP only. Green line is κ-casein and ClpP only. Orange line is κ-casein, BAP and ClpP only. Red line is κ-casein, BAP, ClpP, DnaK, DnaJ and GrpE (KJE). Red line demonstrates almost full degradation of κ-casein after 8 h. (e-g) ATPase and disaggregation activity of ΔN-ClpB pore-loop mutants. (e) ATPase activity assay was carried out according to a recently published protocol (*31*). 1 μM ΔN-ClpB was incubated in the presence of various concentrations of ATP (0 - 3 mM) at 25°C. The initial ATP hydrolysis rate was measured at each ATP concentration using a coupled enzymatic reaction (*31*). Data were then plotted and fitted to Hill equation, to extract the reaction velocity (V_{max}), the half-maximum concentration $K_{0.5}$ and the Hill coefficient n for each measurement. Dots represent the experimental data, and solid lines are the fits. Blue: ΔN-ClpB WT, dark purple: ΔN-ClpB Y243A, light purple: ΔN-ClpB Y643A, brown: ΔN-ClpB Y243A-Y643A. ΔN-ClpB WT data yielded a V_{max} value of 5.1 ± 0.3 min^{-1}, a $K_{0.5}$ value of 300 ± 20 μM and a Hill coefficient of 2.0 ± 0.3. Values for other mutants are listed in Table S12. (f) ATP hydrolysis rate of each ΔN-ClpB mutant with 2 mM ATP (light orange bars) and with 2 mM ATP and 20 μM κ-casein (purple bars) are shown for comparison. (g) G6PDH disaggregation activity assay was conducted similarly to the previously published protocol (*31*). Disaggregation rates were extracted from a time-course experiment and plotted for each ClpB mutant (see Table S12 for tyrosine mutant disaggregation activity). Agg - aggregates only. KJE - aggregates with DnaK, DnaJ and GrpE only. WT - full-length WT ClpB. dN - ΔN-ClpB. S236C, A281C and Y646C are ΔN-ClpB pore-loop single-cysteine mutants (non-labeled). Y1, Y3 and Y1-Y3 are ΔN-ClpB pore-loop tyrosine mutants, Y243A, Y643A and Y243A-Y643A, respectively. S236C-L, A281C-L and Y646C-L are ΔN-ClpB pore-loop single-cysteine mutants, labeled with Alexa 594. All ClpB proteins were incubated with aggregates and the KJE system. The error bars were calculated from two repeats of each experiment. Green dots are actual experimental measurements, while red bars are averages. Errors shown here and in all the other figures correspond to standard errors of the mean.

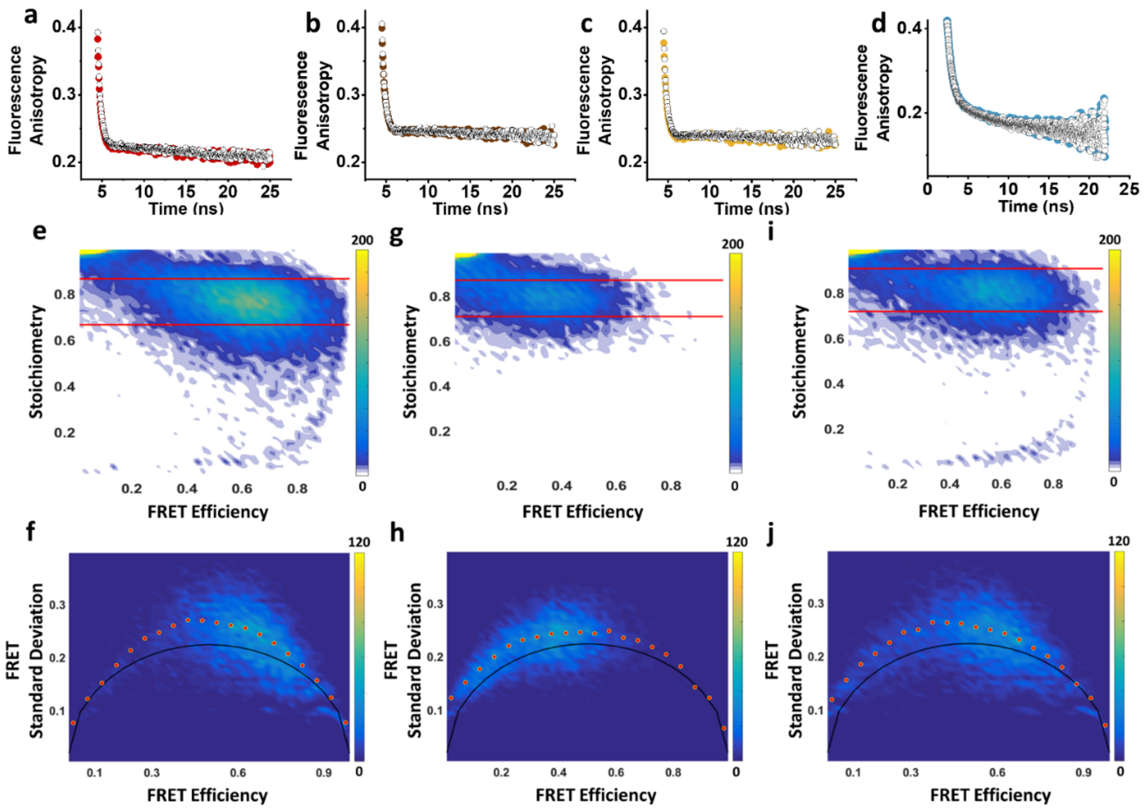

Fig. S2. Analysis of single molecules. (a-d) Time-resolved fluorescence anisotropy measurements of singly labeled pore-loops. Single cysteine variants of ΔN-ClpB were fully labeled with Alexa 488 dye, and then mixed with 1:100 ratio of cysteine-less ΔN-ClpB variant. Mixing and assembly of the complexes were done as described in the Methods section. Samples were diluted to 1-2 nM in the presence of 2 mM ATP, with and without 20 μM κ-casein, and were then measured as described in Methods. (a) ΔN-ClpB S236C with and without κ-casein, shown as black and red dots, respectively. (b) ΔN-ClpB A281C, with (black) and without (brown) κ-casein. (c) ΔN-ClpB Y646C with (black) and without (yellow) κ-casein. (d) ΔN-ClpB S359C with (black) and without (blue) κ-casein. Importantly, no difference in any of the fluorescence anisotropy decays was observed upon the addition of κ-casein. (e-j) 2D FRET-Stoichiometry histograms and burst variance analysis (BVA) plots of ClpB molecules. smFRET experiments with different double-labeled ΔN-ClpB mutants were conducted in the presence of 2 mM ATP. From each experimental data set, a 2D histogram of stoichiometry vs. FRET efficiency was created from ~10,000 burst events as described in the Methods section. The double-labeled species is found at a stoichiometry of 0.6-0.8, the donor-only species at a stoichiometry above 0.96 and the acceptor-only species at a stoichiometry below 0.3. Only bursts of the double-labeled species (marked with red lines) were taken for further analysis (e, g and i). BVA was performed for these selected double-labeled species (*45*) (f, h and j). Black line represents the expected theoretical standard deviation based on shot noise, whereas the blue cloud is the burst-by-burst standard deviation, and the red dots show averaged standard deviation values in bins of size 0.05. The experimental standard deviation values are above the theoretical line, an indication of dynamic heterogeneity. (e, f) ΔN-ClpB (S236C-S359C). (g, h) ΔN-ClpB (A281C-S359C). (i, j) ΔN-ClpB (S359C-Y646C).

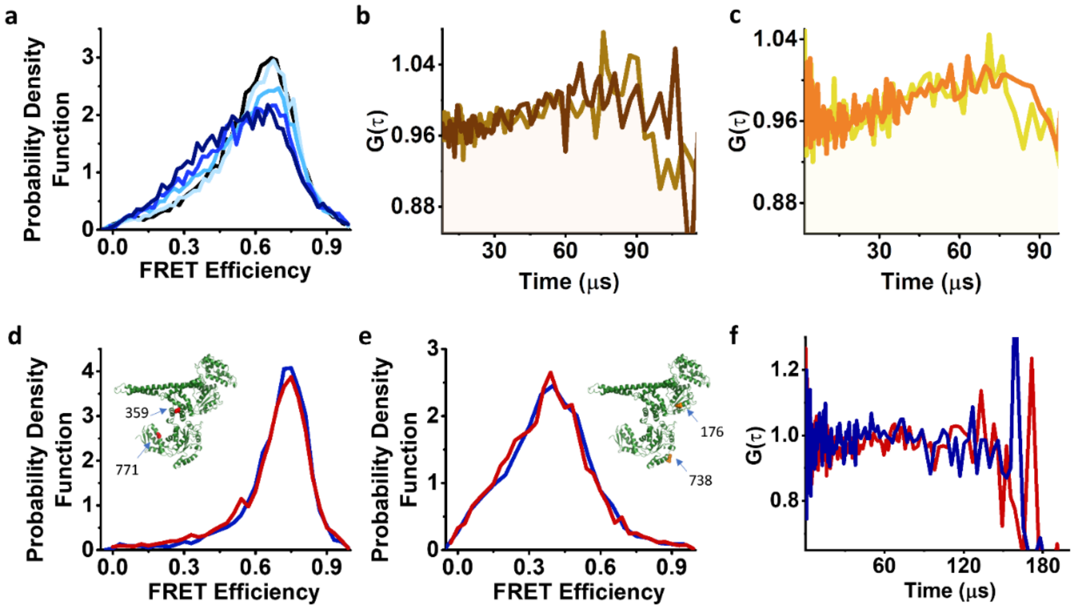

Fig. S3. Additional single-molecule measurements. (a) κ-casein binding to PL1 measured by smFRET. ΔN-ClpB S236C-S359C was incubated with increasing concentrations of κ-casein (0-50 μM), and FRET efficiency histograms were recorded. FRET histograms become broader with increasing κ-casein concentrations. Black curve is at 2 mM ATP and 0 M κ-casein. Light blue to dark blue lines represents the data at 2 mM ATP and 1, 5, 20 and 50 μM κ-casein, respectively. This data set was used to construct the curve in Fig. 2d of the main text. (b-c) Fast dynamics in PL2 and PL3. Donor-acceptor fluorescence cross-correlation functions, calculated from the smFRET measurements of (b) A281C-S359C; light brown- without substrate, dark brown- with substrate and (c) S359C-Y646C; light orange- without substrate, dark orange- with substrate. Both results indicate fast dynamics, on the order of tens of microseconds, with and without substrate. (d-f) NBD1-NBD2 FRET efficiency histograms. (d) ΔN-ClpB was labeled at positions S359C and S771C with Alexa 488 and Alexa 594 fluorescent dyes, and then mixed in 1:100 ratio with the cysteine-less variant. The FRET-efficiency histogram in the presence of 2 mM ATP is shown in blue, and the FRET efficiency histogram in the presence of 2 mM ATP and 20 μM κ-casein is shown in red. (e) Another probe of NBD1-NBD2 dynamics, using the pair D176C-Q738C. Color code is the same as in (d). From both panels, we can conclude that there is no effect of κ-casein on NBD1-NBD2 conformational dynamics. (f) Donor-acceptor cross-correlation curves of the construct D176C-Q783C, with (red) and without (blue) κ-casein, do not detect any NBD1-NBD2 interdomain dynamics on the microsecond time scale.

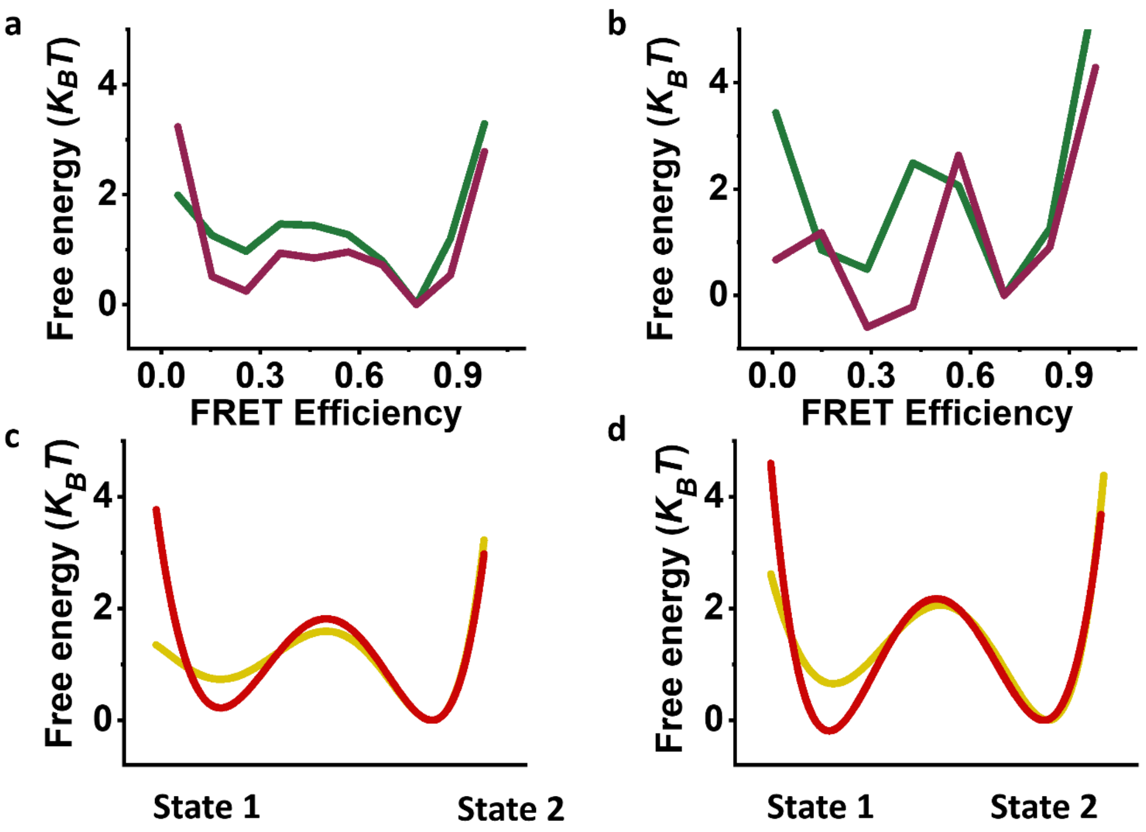

Fig. S4. Energy landscape as obtained from H^{2}MM analysis. (a-b) Line plots of the free-energy profiles of pore loops, as obtained from H^{2}MM analysis. Pore-loop FRET data measured in the presence of 2 mM ATP and either without (green line) or with 20 µM κ-casein (purple line), were analyzed as discussed in Methods, using 9 sequentially connected states. The free-energy profile was calculated from state propensities obtained from this model. (a) PL1 (ΔN-ClpB S236C-S359C). (b) PL3 (ΔN-ClpB S359C-Y646C). The two well-defined minima suggest that a two-state model is appropriate to describe the data, as in the case of PL2 (Fig. 3 of the main text). (c-d) Line plots of the effective free-energy profiles as obtained from H^{2}MM analysis using a two-state model, in the presence of 2 mM ATP and either without (yellow line) or with 20 µM κ-casein (red line). The free-energy profiles were calculated using H^{2}MM parameters as indicated in Tables S3-S5. The barrier heights were calculated using the Arrhenius equation with a pre-exponential factor of 10^5 s^{-1}. (c) PL1 (ΔN-ClpB S236C-S359C) and (d) PL3 (ΔN-ClpB S359C-Y646C).

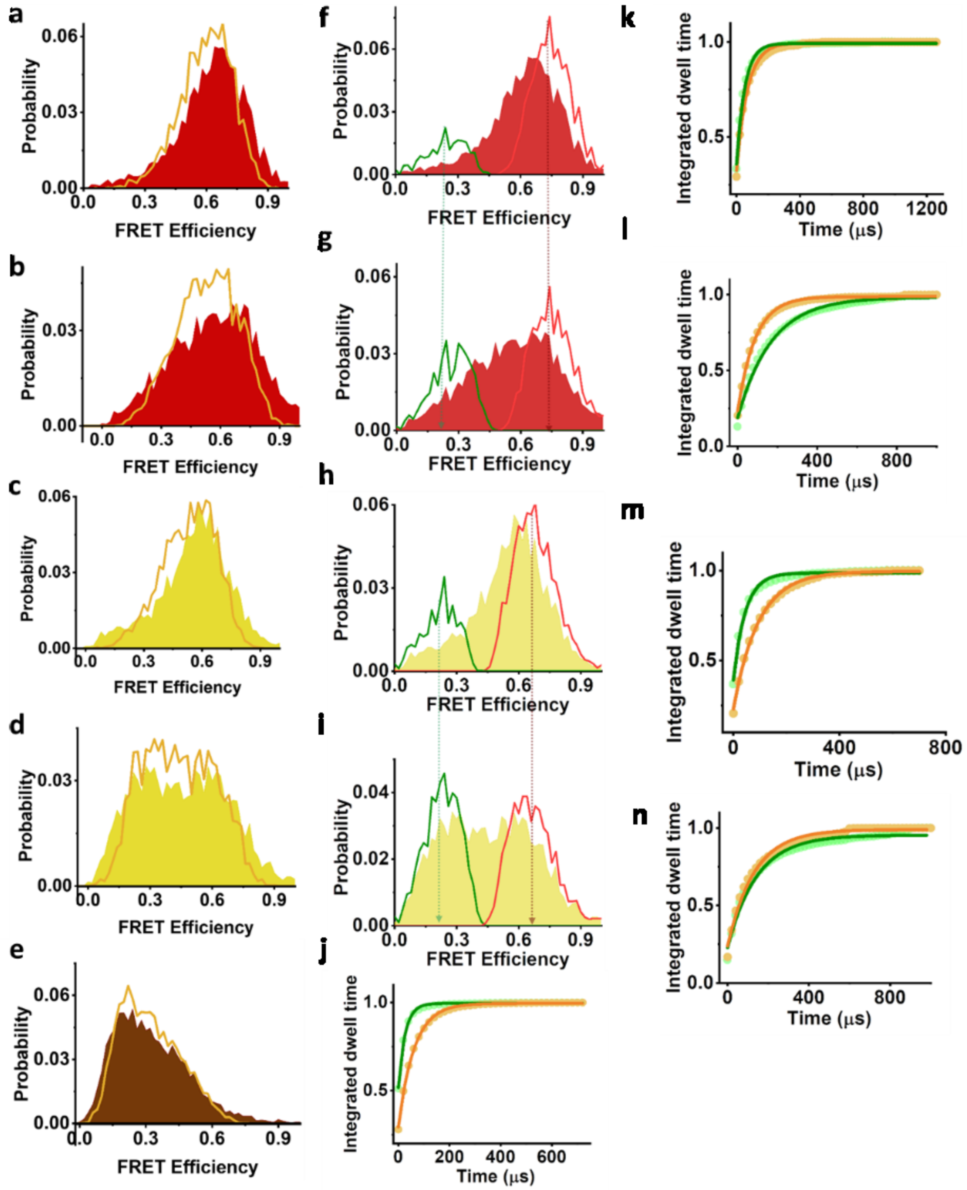

Fig. S5. H²MM data analysis. (a-e) Recoloring of FRET-efficiency histograms. FRET-efficiency histograms were recolored based on the results of H²MM analysis with a two-state model, following the procedure outlined in the Methods section of the main text and Ref. (*31*). Recolored histograms are shown as orange lines. (a-b) ΔN-ClpB S236C-S359C, without and with 20 μM κ-casein, respectively. (c-d) ΔN-ClpB S359C-Y646C, without and with 20 μM κ-casein, respectively. (e) ΔN-ClpB A281C-S359C, with 20 μM κ-casein. (f-i) Segmentation analysis. Following the two-state H²MM analysis, FRET-efficiency histograms were segmented according to the procedure outlined in the Methods section and Ref. (*31*) in order to obtain the distributions of the separate states. In each histogram, green line is the low FRET population, and red line is the high FRET population, as obtained from the segmentation analysis. Arrows point to FRET-efficiency values of the two states obtained from the analysis. (f-g) ΔN-ClpB S236C-S359C, without and with 20 μM κ-casein, respectively. (h-i) ΔN-ClpB S359C-Y646C, without and with 20 μM κ-casein, respectively. In all pore-loop data, an increase in low FRET population was observed in the

presence of 20 μM κ-casein. H^2MM model parameters are listed in Tables S3-S5. (j-n) Dwell time analysis. Integrated dwell-time distributions were calculated based on the results of the two-state H^2MM analysis, according to the procedure described in Ref. (*44*). State 1 is shown as green dots, and state 2 is shown as orange dots. Green and orange solid lines are fits to single-exponential functions, and the obtained rates are listed in Table S3. (j-k) S236C-S359C without and with 20 μM κ-casein, respectively. (l) A281C-S359C with 20 μM κ-casein. (m-n) S359C-Y646C without and with 20 μM κ-casein, respectively.

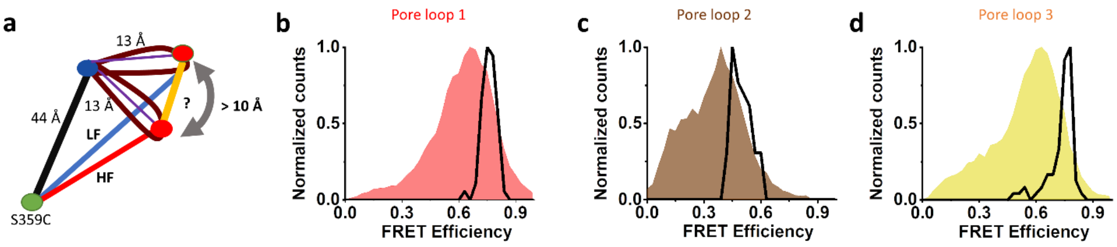

Fig. S6. Pore-loop conformational space as obtained from smFRET experiments and predicted from cryo-EM data. (a) Triangulation calculation to obtain the approximate movements of PL1 (thick purple curves) vertically along the axial channel (orange line), around their pivot point (blue dot). Purple lines represent the approximate distance between the labeled position on PL1 and the pivot point, assumed to be fixed at 13 Å. Black line represents the distance of the pivot point to S359C, which is also assumed to be fixed at 44 Å. Blue and red lines are the extracted distances of the experimental high FRET-efficiency and low FRET-efficiency states, respectively. With the given distances, we calculated a distance of more than 10 Å (~ 2 amino acids), as the possible amplitude of pore-loop motion along the axial channel (vertically). This result, and similar calculations for PL2 and PL3, indicate that measured pore-loop dynamics represent functionally significant fluctuations. The fixed distances were obtained from a cryo-EM structure of ClpB (PDB: 5og1, protomer F) (*14*). The calculation does not take into account the contribution of dye linkers to the distance changes, and may therefore slightly overestimate the amplitude of motion. (b-d) Pore-loop distances (S236C, A281C, Y646C) were measured relative to our reference point (S359C), from various available cryo-EM structures of *E. coli* ClpB (*5, 14, 19*). Using a Förster distance of 54 Å, we converted these distances to FRET efficiencies. The resulting 'theoretical' FRET-efficiency distributions are plotted as black lines, together with the experimental FRET-efficiency distributions (area plots) for each pore loop, for comparison. (b) S236C-S359C. (c) A281C-S359C. (d) S359C-Y646C. The experimental smFRET histograms are much broader than obtained from cryo-EM structures, indicating that the pore loops sample a much larger conformational space than can be gleaned from frozen structures.

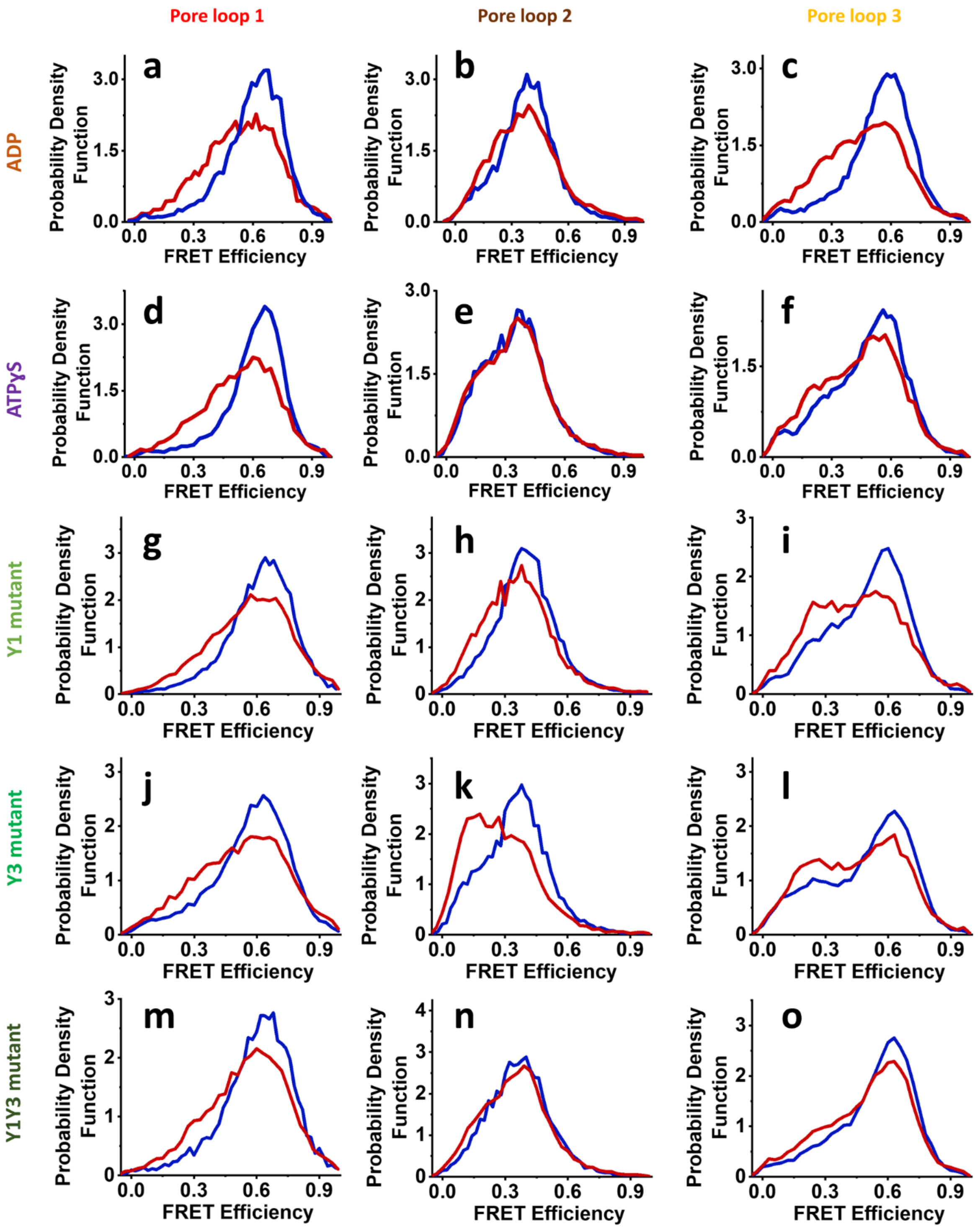

Fig. S7. The effects of different nucleotides and tyrosine mutations on the induction of conformational changes of pore loops by a protein substrate. FRET-efficiency histograms were measured for the three pore loops (PL1-PL3) under a series of conditions as indicated. Left column- PL1. Middle column- PL2, right column- PL3. In each panel, blue line is without substrate and red line is with 20 μM κ-casein. (a-c) 2 mM ADP. (d-f) 2 mM ATPγS. (g-i) Y1 mutant. (j-l) Y3. (m-o) Y1-Y3.

Table S1. Sequence of ClpB and list of primers for all ClpB mutants.

```
TT 1 MNLERWTQAAR-E-ALAQAQVLAQRMKH--QAIDLP-HLW-AVLLKDE-RSLAWRLLEKA 53
EC 1 MRLDRLTN--KFQLALADAQSLA--LGHDNQFIE-PLHLMSA-LLNQEGGSVS-PLLTSA 53

TT 54 G--ADPKALK-EL-QERELARLPKVE--GAEVGQYLTSR-L-SGALNRAEALMEELK-DR 104
EC 54 GINAG-Q-LRTDINQA--LNRLPQVEGTGGDV-Q--PSQDLVR-VLNLCDKLAQK-RGDN 104

TT 105 YVAVDTL-VLALA-EATPG-LPG-LEALKG---A-L-K--E-LRGGRTVQTEHAE-STYN 151
EC 105 FISSE-LFVLA-ALESR-GTLADILKA-AGATTANITQAIEQMRGGESVNDQGAEDQR-Q 159

TT 152 ALEQYGIDLT-RLAAE-GKLDPVIGRDEEIRRVIQILLRRTKNNPVLIGEPGVGKTAIVE 209
EC 160 ALKKYTIDLTER-A-EQGKLDPVIGRDEEIRRTIQVLQRRTKNNPVLIGEPGVGKTAIVE 217

TT 210 GLAQRIVKGDVPEGLKGKRIVSLQMGSLLAGAKYRGEFEERLKAVIQEVV-QSQGEVILF 268
EC 218 GLAQRIINGEVPEGLKGRRVLALDMGALVAGAKYRGEFEERLKGVLNDLAKQ-EGNVILF 276

TT 269 IDELHTVVGAGKAEGAVDAGNMLKPALARGELRLIGATTLDEYRE-IEKDPALERRFQPV 327
EC 277 IDELHTMVGAGKADGAMDAGNMLKPALARGELHCVGATTLDEYRQYIEKDAALERRFQKV 336

TT 328 YVDEPTVEETISILRGLKEKYEVHHGVRISDSAIIAAATLSHRYITERRLPDKAIDLIDE 387
EC 337 FVAEPSVEDTIAILRGLKERYELHHHVQITDPAIVAAATLSHRYIADRQLPDKAIDLIDE 396

TT 388 AAARLRMALESAPEEIDALERKKLQLEIEREALKKEKDPD-SQERLKAI-EAEIA-KLTE 444
EC 397 AASSIRMQIDSKPEELDRLDRRIIQLKLEQQALMKESD-EASKKRLDMLNE-ELSDK--E 452

TT 445 -EIAKLRAEWERER-EIL---RKLREAQHRLDEVRREIELAERQY-DLNRAAELRYGELP 498
EC 453 RQYSELEEEWKAEKAS-LSGTQTIK-AE--LEQAKIAIEQA-RRVGDLARMSELQYGKIP 507

TT 499 KLEAEVEALSEKLRG-A-RFVR-LEVTE-EDIAEIVSRWTGIPVSKLLEGEREKLLRLEE 554
EC 508 ELEKQLEAATQ-LEGKTMRLLRN-KVTDAE-IAEVLARWTGIPVSRMMESEREKLLRMEQ 564

TT 555 ELHKRVVGQDEAIRAVADAIRRARAGLKDPNRPIGSFLFLGPTGVGKTELAKTLAATLFD 614
EC 565 ELHHRVIGQNEAVDAVSNAIRRSRAGLADPNRPIGSFLFLGPTGVGKTELCKALANFMFD 624

TT 615 TEEAMIRIDMTEYMEKHAVSRLIGAPPGYVGYEEGGQLTEAVRRRPYSVILFDEIEKAHP 674
EC 625 SDEAMVRIDMSEFMEKHSVSRLVGAPPGYVGYEEGGYLTEAVRRRPYSVILLDEVEKAHP 684

TT 675 DVFNILLQILDDGRLTDSHGRTVDFRNTVIILTSNLGSPLILE--G-LQKGWPYERIRDE 731
EC 685 DVFNILLQVLDDGRLTDGQGRTVDFRNTVVIMTSNLGSDLIQERFGELD----YAHMK-E 739

TT 732 -VFKVLQQH-FRPEFLNRLDEIVVFRPLTKEQ-IRQIVEIQL-S-YLRARLAEKR-ISLE 785
EC 740 LVLGVVS-HNFRPEFINRIDEVVVFHPLG-EQHIASIAQIQLKRLY-K-RL-EERGYEIH 794

TT 786 LT-EAAKDFLAERGYDPVFGARPLRRVIQRELETPLAQKILAGE-VKEGDRV-QVDVGPA 842
EC 795 ISDEALK-LLSENGYDPVYGARPLKRAIQQQIENPLAQQILSGELVP-G-KVIRLEVNED 851

TT 843 GLVFAV-PARVEA 854
EC 852 RIV-AVQ------ 857
```

Name	Sequence
**S236C**	5’ gtctccttgcagatgggc**tgc**ctcctcgccggggccaag 3’
**L238C**	5’ ccttgcagatgggctccctc**tgc**gccggggccaagtaccgggg 3’
**A239C**	5’ cagatgggctccctcctc**tgc**ggggccaagtaccg 3’
**Y243A (PL1 tyrosine mutant)**	5’ cctcgccggggccaag**gcc**cggggcgagtttgaggagc 3’

Name	Sequence
**A281C**	5’ gtggtgggggcaggcaag**tgc**gagggcgccgtggacgcg 3’
**S359C**	5’ gtgcgcatctccgac**tgc**gccatcatcgccgcc 3’
**Y643A (PL3 tyrosine mutant)**	5’ cggggccccgcccggc**gcc**gtgggctacgaggagggggggg 3’
**Y643A (PL3 tyrosine mutant) on Y646C mutant**	5’ cggggccccgcccggc**gcc**gtgggc**tgc**gaggaggggggggg 3’
**Y646C**	5’ ccccgcccggctacgtgggc**tgc**gaggaggggggggcagctca 3’

The first part of this table shows sequence alignment of *TT* ClpB with *E. coli* (EC) ClpB. PL1 labeling site is marked by a red rectangle, PL2 labeling site is marked by a brown rectangle and PL3 labeling site is marked by an orange rectangle. Tyrosine mutants are marked with green rectangles. S359 is highlighted in orange. Note that in all experiments in this study, ΔN-ClpB of *TT* ClpB without the first 140 residues was used, the start of which is marked with a black box. The alignment was done using Sequence Manipulation Suite, Pairwise Align Protein Tool, and using scoring matrix BLOSUM 80. The second part includes all primers used in this work.

Table S2. Photophysical properties of fluorescent dyes attached to ClpB.

Construct	Steady-state anisotropy	Steady-state anisotropy calculated from time-resolved measurements	Quantum yield
**S236C + Alexa 488 (ATP)**	0.307 ± 0.004	0.278 ± 0.002	0.71 ± 0.06
**S236C + Alexa 488 (ATP + κ-casein)**	0.303 ± 0.007	0.276 ± 0.002	-
**A281C + Alexa 488 (ATP)**	0.263 ± 0.004	0.277 ± 0.002	0.72 ± 0.08
**A281C + Alexa 488 (ATP + κ-casein)**	0.264 ± 0.003	0.275 ± 0.002	-
**Y646C + Alexa 488 (ATP)**	0.235 ± 0.003	0.25 ± 0.001	0.71 ± 0.05
**Y646C + Alexa 488 (ATP + κ-casein)**	0.249 ± 0.004	0.25 ± 0.001	-
**S359C + Alexa 488 (ATP)**	0.205 ± 0.003	0.20 ± 0.02	N.D.
**S359C + Alexa 488 (ATP + κ-casein)**	0.203 ± 0.001	0.20 ± 0.05	-

Table S3. State-to-state transition rates (in s^{-1}) obtained from H^2MM analysis of ClpB constructs, compared to dwell times obtained as in Fig. S5.

	k_{12}		k_{21}		$k_{12}+k_{21}$	
**Construct**	**H^2MM**	**Dwell time**	**H^2MM**	**Dwell time**	**H^2MM**	**Dwell time**
**PL1**	45960 ± 5100	37660 ± 1100	22130 ± 2500	16300 ± 220	68090 ± 7600	53960 ± 1320
**PL1 + κ-casein**	21450 ± 1600	19300 ± 500	17500 ± 1700	14500 ± 300	38950 ± 3300	33800 ± 800
**PL2**	12300 ± 750	10300 ± 300	9580 ± 200	8750 ± 100	21880 ± 950	19050 ± 400
**PL2 + κ-casein**	6500 ± 800	5900 ± 200	11200 ± 1700	11300 ± 300	17700 ± 2500	17200 ± 500
**PL3**	24500 ± 100	22200 ± 950	12800 ± 100	10600 ± 200	37300 ± 200	32800 ± 1150
**PL3 + κ-casein**	8700 ± 1000	6400 ± 300	10400 ± 1500	7500 ± 200	19100 ± 2500	13900 ± 500

The errors were calculated from two independent repeats of the experiment.

Table S4. FRET efficiency values of pore loop states as obtained from H^2MM analysis.

**Pore loop**	**FRET value** State 1	**FRET value** State 2
**PL1**	0.240 ± 0.005	0.76 ± 0.005
**PL2**	0.180 ± 0.005	0.54 ± 0.005
**PL3**	0.210 ± 0.005	0.68 ± 0.005

Table S5. State populations of pore loops from experiments with different nucleotides and either with or without the substrate, as obtained from H^2MM analysis.

	**ATP**		**ADP**		**ATPγS**	
**Pore loop**	**State 1**	**State 2**	**State 1**	**State 2**	**State 1**	**State 2**
**PL1**	0.32 ±0.01	0.68 ± 0.01	0.30 ± 0.02	0.70 ± 0.02	0.30 ± 0.01	0.70 ±0.01
**PL1 + κ-casein**	0.45 ±0.01	0.55 ± 0.01	0.44 ± 0.02	0.56 ± 0.02	0.44 ± 0.02	0.56 ±0.02
**PL2**	0.43 ±0.01	0.57 ± 0.01	0.37 ± 0.02	0.63 ± 0.02	0.50 ± 0.01	0.50 ±0.01
**PL2 + κ-casein**	0.63 ±0.01	0.37 ± 0.01	0.51 ± 0.01	0.49 ± 0.01	0.50 ± 0.01	0.50 ±0.01
**PL3**	0.34 ±0.01	0.66 ± 0.01	0.31 ± 0.01	0.69 ± 0.01	0.40 ± 0.01	0.60 ±0.01
**PL3 + κ-casein**	0.54 ±0.01	0.46 ± 0.01	0.44 ± 0.01	0.56 ± 0.01	0.47 ± 0.01	0.53 ±0.01

The errors were calculated from two independent repeats of the experiment.

Table S6. State populations of pore loops in tyrosine mutants with and without substrate, as obtained from H^2MM analysis.

	WT		Y1		Y3		Y1-Y3	
**Pore loop**	**State 1**	**State 2**	**State 1**	**State 2**	**State 1**	**State 2**	**State 1**	**State 2**
**PL1**	0.32 ±0.01	0.68 ± 0.01	0.27 ± 0.02	0.73 ± 0.02	0.35 ± 0.01	0.65 ± 0.01	0.30 ± 0.01	0.70 ± 0.01
**PL1 + κ-casein**	0.45 ± 0.01	0.55 ± 0.01	0.37 ± 0.02	0.63 ± 0.02	0.45 ± 0.02	0.55 ± 0.02	0.38 ± 0.01	0.62 ± 0.01
**PL2**	0.43 ± 0.01	0.57 ± 0.01	0.37 ± 0.02	0.63 ± 0.02	0.46 ± 0.01	0.54 ± 0.01	0.44 ± 0.01	0.56 ± 0.01
**PL2 + κ-casein**	0.63 ± 0.01	0.37 ± 0.01	0.44 ± 0.01	0.56 ± 0.01	0.60 ± 0.01	0.40 ± 0.01	0.47 ± 0.01	0.53 ± 0.01
**PL3**	0.34 ± 0.01	0.66 ± 0.01	0.38 ± 0.01	0.62 ± 0.01	0.40 ± 0.01	0.60 ± 0.01	0.30 ± 0.01	0.70 ± 0.01
**PL3 + κ-casein**	0.54 ± 0.01	0.46 ± 0.01	0.52 ± 0.01	0.48 ± 0.01	0.50 ± 0.01	0.50 ± 0.01	0.37 ± 0.01	0.63 ± 0.01

The errors were calculated from two independent repeats of the experiment.

Table S7. Equilibrium constants, K_{12}^{i}, for pore-loop conformational dynamics as obtained from H^2MM analysis in the presence of different nucleotides.

	Nucleotide			Nucleotide + κ-casein		
**Pore loop**	**ATP**	**ADP**	**ATPγS**	**ATP**	**ADP**	**ATPγS**
**PL1**	0.48 ± 0.01	0.45 ± 0.02	0.46 ± 0.02	0.81 ± 0.01	0.77 ± 0.02	0.78 ± 0.02
**PL2**	0.76 ± 0.03	0.59 ± 0.01	0.97 ± 0.01	1.70 ± 0.04	0.99 ± 0.06	0.97 ± 0.01
**PL3**	0.52 ± 0.01	0.45 ± 0.01	0.69 ± 0.02	1.19 ± 0.02	0.77 ± 0.02	0.90 ± 0.09

The errors were calculated from two independent repeats of the experiment.

Table S8. Equilibrium constants, K_{12}^{i}, from experiments with tyrosine mutants as obtained from H^2MM analysis.

	ATP				ATP + κ-casein			
**Pore loop**	**WT**	**Y1**	**Y3**	**Y1-Y3**	**WT**	**Y1**	**Y3**	**Y1-Y3**
**PL1**	0.48 ± 0.01	0.37 ± 0.01	0.53 ± 0.02	0.42 ± 0.01	0.81 ± 0.01	0.59 ± 0.02	0.81 ± 0.01	0.61 ± 0.01
**PL2**	0.76 ± 0.03	0.58 ± 0.02	0.86 ± 0.03	0.77 ± 0.02	1.70 ± 0.04	0.80 ± 0.03	1.47 ± 0.02	0.87 ± 0.03
**PL3**	0.52 ± 0.01	0.61 ± 0.01	0.63 ± 0.03	0.44 ± 0.02	1.12 ± 0.02	1.07 ± 0.01	0.95 ± 0.03	0.58 ± 0.02

The errors were calculated from two independent repeats of the experiment.

Table S9. State-to-state transition rates (in s^{-1}) obtained from H^2MM analysis of ClpB constructs in the presence of different nucleotides.

	ATP		ADP		ATPγS	
**Construct**	k_{12}	k_{21}	k_{12}	k_{21}	k_{12}	k_{21}
**PL1**	45960 ± 5100	22130 ± 2500	44800 ± 1700	19800 ± 600	42500 ± 9000	18500 ± 600
**PL1 + κ-casein**	21450 ± 1600	17500 ± 1700	24000 ± 500	18000 ± 100	22500 ± 3000	17500 ± 1100
**PL2**	12300 ± 750	9580 ± 200	22800 ± 1100	13500 ± 300	12600 ± 300	12400 ± 200
**PL2 + κ-casein**	6500 ± 800	11200 ± 1700	12700 ± 1900	13500 ± 2800	11800 ± 900	11500 ± 1100
**PL3**	24500 ± 100	12800 ± 100	33400 ± 800	15300 ± 100	23500 ± 3000	15600 ± 2500
**PL3 + κ-casein**	8700 ± 1000	10400 ± 1500	18000 ± 300	14000 ± 200	15500 ± 1300	14000 ± 2500

The errors were calculated from two independent repeats of the experiment.

Table S10. State-to-state transition rates (in s^{-1}) obtained from H^2MM analysis of ClpB tyrosine mutants.

	WT		Y1		Y3		Y1-Y3	
**Construct**	k_{12}	k_{21}	k_{12}	k_{21}	k_{12}	k_{21}	k_{12}	k_{21}
**PL1**	45960 ± 5100	22130 ± 2500	48300 ± 1000	18200 ± 2500	35000 ± 1800	18700 ± 500	48800 ± 900	20000 ± 200
**PL1 + κ-casein**	21450 ± 1600	17500 ± 1700	29400 ± 1300	17500 ± 1700	20500 ± 300	16700 ± 500	27600 ± 300	16800 ± 200
**PL2**	12300 ± 750	9580 ± 200	26300 ± 300	15400 ± 100	17100 ± 200	14600 ± 500	20400 ± 300	16000 ± 100
**PL2 + κ-casein**	6500 ± 800	11200 ± 1700	17100 ± 200	13700 ± 200	8500 ± 300	12600 ± 500	14600 ± 200	12800 ± 100
**PL3**	24500 ± 100	12800 ± 100	23300 ± 800	14400 ± 600	15300 ± 300	10000 ± 100	23000 ± 500	10300 ± 100
**PL3 + κ-casein**	8700 ± 1000	10400 ± 1500	14200 ± 200	15300 ± 400	10900 ± 700	10500 ± 500	18100 ± 400	10500 ± 300

The errors were calculated from two independent repeats of the experiment.

Table S11. Substrate-response factors, R_i, as obtained from H^2MM analysis.

	Nucleotide			Tyrosine mutants		
**Pore loop**	**ATP**	**ADP**	**ATPγS**	**Y1**	**Y3**	**Y1-Y3**
**PL1**	1.68 ± 0.01	1.71 ± 0.02	1.69 ± 0.02	1.59 ± 0.02	1.52 ± 0.01	1.46 ± 0.01
**PL2**	2.25 ± 0.03	1.67 ± 0.02	1.00 ± 0.02	1.39 ± 0.02	1.76 ± 0.01	1.12 ± 0.05
**PL3**	2.28 ± 0.01	1.71 ± 0.02	1.34 ± 0.09	1.76 ± 0.01	1.50 ± 0.02	1.23 ± 0.05

The errors were calculated from two independent repeats of the experiment.

Table S12. Enzymatic activity parameters of ΔN-ClpB and its pore loop mutants.

ClpB variant	V_{max} (1/min)	$K_{0.5}$ (µM)	n	Dissaggregation rate (nM/min)
**WT**	5.1 ± 0.3	300 ± 20	2.0 ± 0.2	3.89 ± 0.43
**Y1**	4.6 ± 0.1	405 ± 30	2.2 ± 0.2	2.35 ± 0.39
**Y3**	2.2 ± 0.1	430 ± 40	2.7 ± 0.6	1.07 ± 0.33
**Y1-Y3**	2.2 ± 0.1	730 ± 50	2.0 ± 0.2	0.3 ± 0.32

Parameters obtained from fits of ATPase activity of ΔN-ClpB and its pore loop mutants to the Hill equation, see the Methods section "ATPase activity". Errors were obtained from two repeats of the experiment.
